# Madeng’ai Ethyl Acetate Extract Attenuates Lipopolysaccharide-Induced Inflammation and Acute Lung Injury

**DOI:** 10.3390/ijms27219430

**Published:** 2026-10-22

**Authors:** Yanbin Zhang, Wenbin Ji, Liang Cao, Yan Wang, Wei Wang, Zaiqi Zhang

**Affiliations:** 1 The First Clinical Medical College, Ningxia Medical University, Yinchuan 750004, China; yanbin202410@163.com (Y.Z.); jiwb@nxmu.edu.cn (W.J.); 2 Hunan Provincial Key Laboratory of Dong Medicine, Hunan University of Medicine, Huaihua 418000, China; caoliang520945@126.com; 3 College of Biological Resources and Environmental Sciences, Jishou University, Jishou 416000, China; wangzhongwang306@outlook.com; 4 Traditional Chinese Medicine (TCM) and Ethnomedicine Innovation & Development International Laboratory, School of Pharmacy, Hunan University of Chinese Medicine, Changsha 410208, China

**Keywords:** *Potentilla freyniana*, acute lung injury, NLRP3 inflammasome, gasdermin D, oxidative stress, network pharmacology

## Abstract

Madeng’ai, the dried root of *Potentilla freyniana* Bornm., is used in Dong medicine for infections and wound care. This study examined the chemical composition and prophylactic protective effects of the ethyl acetate fraction of its ethanolic root extract (referred to as MEA) in lipopolysaccharide (LPS)-induced acute lung injury (ALI). Ultra-high-performance liquid chromatography coupled to Q-Exactive Orbitrap mass spectrometry detected 77 constituents, comprising 13 compounds authenticated with reference standards and 64 constituents tentatively annotated from accurate mass and tandem mass spectrometry (MS/MS) fragmentation. Network pharmacology prioritized six candidate compounds, 120 overlapping targets, and nine hub targets, and molecular docking explored theoretical interactions with inflammation-related proteins. MEA was evaluated in LPS-stimulated MH-S alveolar macrophages and in male C57BL/6J mice receiving oral pretreatment for seven days prior to intraperitoneal LPS challenge. In MH-S cells, MEA pretreatment reduced nitrite, interleukin (IL)-1β, IL-6, tumor necrosis factor (TNF)-α, 2′,7′-dichlorodihydrofluorescein diacetate (DCFH-DA)-detectable intracellular oxidative fluorescence, and *Nlrp3*, *Pycard*, *Casp1*, and *Gsdmd* mRNA levels. In mice, seven-day oral pretreatment with MEA attenuated qualitative histological lung injury, pulmonary edema, and serum cytokine elevation, accompanied by lower lung protein levels of NOD-like receptor family pyrin domain-containing 3 (NLRP3), apoptosis-associated speck-like protein containing a CARD (ASC), pro-caspase-1, and the ~30 kDa N-terminal fragment of gasdermin D (GSDMD-N), together with related transcripts. These findings indicate that prophylactic MEA administration attenuates LPS-induced macrophage inflammation and experimental lung injury, accompanied by reduced intracellular oxidative stress and decreased expression of NLRP3–GSDMD pathway-associated markers.

## 1. Introduction

Acute lung injury (ALI) is characterized by inflammatory damage to the alveolar epithelium and pulmonary capillary endothelium. Disruption of this barrier promotes alveolar and interstitial edema, impairs gas exchange, and contributes to acute respiratory distress syndrome (ARDS) [1,2]. Supportive care remains central to management, while pharmacological control of the underlying inflammatory injury remains challenging. The interaction between inflammatory responses and barrier dysfunction makes pulmonary inflammation an important focus of experimental ALI research.

The NOD-like receptor family pyrin domain-containing 3 (NLRP3) inflammasome connects innate immune sensing with inflammatory cell injury. It comprises the NLRP3 sensor, the adaptor apoptosis-associated speck-like protein containing a CARD (ASC), and pro-caspase-1 [3,4]. Inflammasome assembly facilitates caspase-1 activation, cytokine maturation, and gasdermin D (GSDMD) cleavage. The resulting N-terminal fragment, GSDMD-N, forms membrane pores that support cytokine release and pyroptotic cell death [5,6]. In experimental ALI, excessive NLRP3 signaling contributes to macrophage inflammatory responses and pulmonary barrier injury [7,8]. This pathway therefore offers a biological focus for investigating agents that attenuate lung inflammation.

Plants of the genus *Potentilla* (Rosaceae) are rich sources of polyphenols, flavonoids, and triterpenoids with documented anti-inflammatory, antioxidant, and antimicrobial activities [9]. *Potentilla freyniana* Bornm., whose dried root is known as Madeng’ai in traditional Dong medicine in China, has long been used ethnomedicinally for bacterial infections, traumatic bleeding, and inflammatory wound care [10]. Phytochemical investigations of *P. freyniana* have identified diverse secondary metabolites, including flavan-3-ols, oligomeric procyanidins, flavonols, dihydrochalcones, hydrolysable tannins, and ursane- or oleanane-type pentacyclic triterpenoids such as rosamultin, pomolic acid, and ursolic acid [11,12]. Preclinical studies have reported that extracts and constituents of *P. freyniana* exhibit antimicrobial activity [10], selective cytotoxicity [12,13], and anti-inflammatory efficacy in experimental gouty arthritis via modulation of mitogen-activated protein kinase (MAPK)/nuclear factor-κB (NF-κB)/NLRP3 signaling [14], although clinical studies have not yet been conducted. While our recent integrated multi-omics study showed that the ethyl acetate fraction of Madeng’ai mitigated lipopolysaccharide (LPS)-induced ALI in mice at the level of global metabolic and transcriptomic remodeling [15], its reference-standard-authenticated chemical profile, its direct anti-inflammatory and redox effects in alveolar macrophages, and its impact on pulmonary NLRP3–GSDMD pathway-related marker expression remained uncharacterized. In the present study, using an independent experimental dataset, we authenticated 13 constituents among 77 ultra-high-performance liquid chromatography coupled to Q-Exactive Orbitrap mass spectrometry (UHPLC-Q-Exactive Orbitrap MS) peaks using reference standards, prioritized candidate targets via network pharmacology and molecular docking, and evaluated whether prophylactic administration of the ethyl acetate fraction of the ethanolic root extract of Madeng’ai (MEA) attenuates LPS-induced oxidative and inflammatory responses in MH-S alveolar macrophages and downregulates NLRP3–GSDMD pathway-associated markers in murine ALI.

## 2. Results

### 2.1. Chemical Composition of MEA

**Figure 1:**
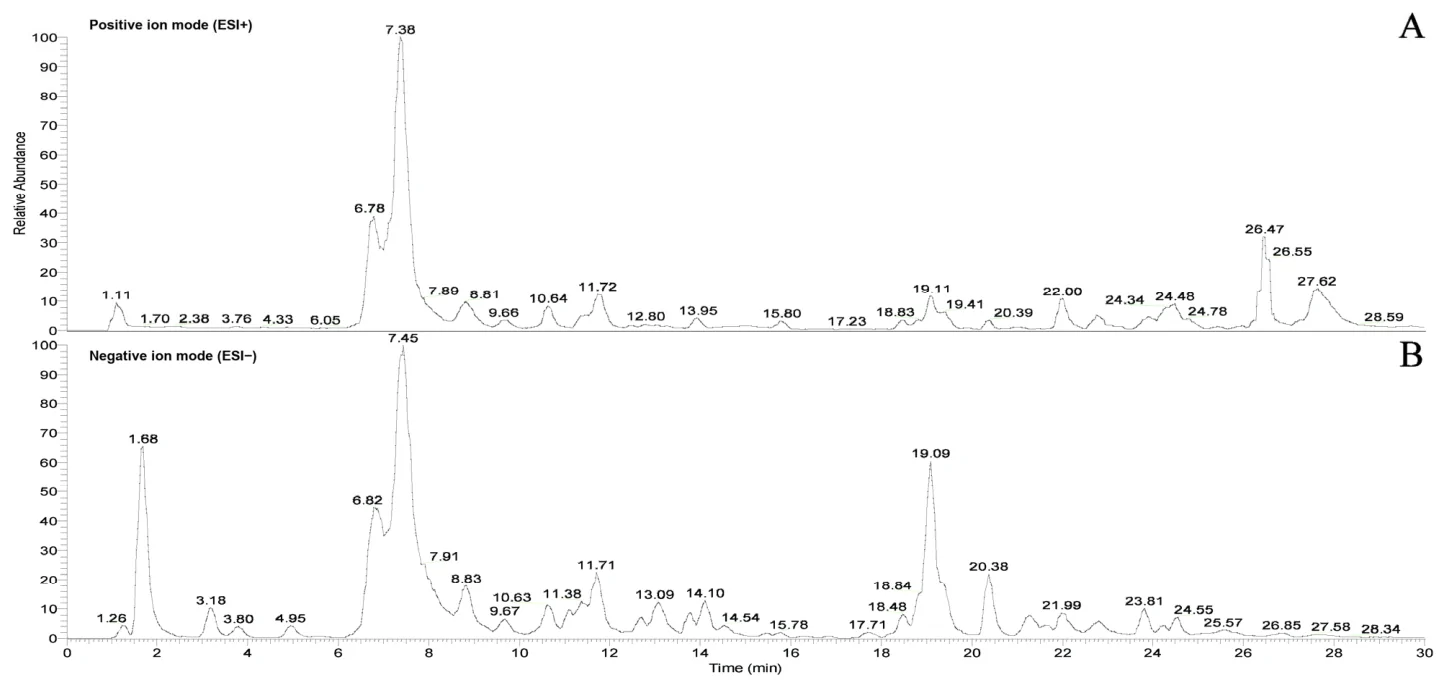
Base peak chromatograms of MEA acquired by UHPLC-Q-Exactive Orbitrap MS in (**A**) positive and (**B**) negative ionization modes. Peaks correspond to constituents listed in Table 1 and Supplementary Table S1.

UHPLC-Q-Exactive Orbitrap MS detected 77 constituents in MEA, including 34 flavonoids, 28 tannins, 10 triterpenoids, four organic acids, and one phytosterol (Supplementary Table S1). Among these, 13 compounds were unambiguously confirmed by matching retention times, accurate mass, and tandem mass spectrometry (MS/MS) spectra with authentic reference standards: gallic acid, procyanidin B1, catechin, taxifolin, epicatechin gallate, ellagic acid, phlorizin, quercetin, naringenin, phloretin, rosamultin, pomolic acid, and ursolic acid (Table 1). The remaining 64 constituents were tentatively annotated on the basis of accurate mass and MS/MS fragmentation patterns without reference-standard verification. This qualitative LC-MS analysis was designed for structural profiling and reference-standard authentication; absolute concentrations and relative peak area percentages of individual constituents in MEA were not quantified. Base peak chromatograms in positive and negative ion modes are shown in Figure 1.

### 2.2. Candidate Compounds and MEA-ALI Targets

**Figure 2:**
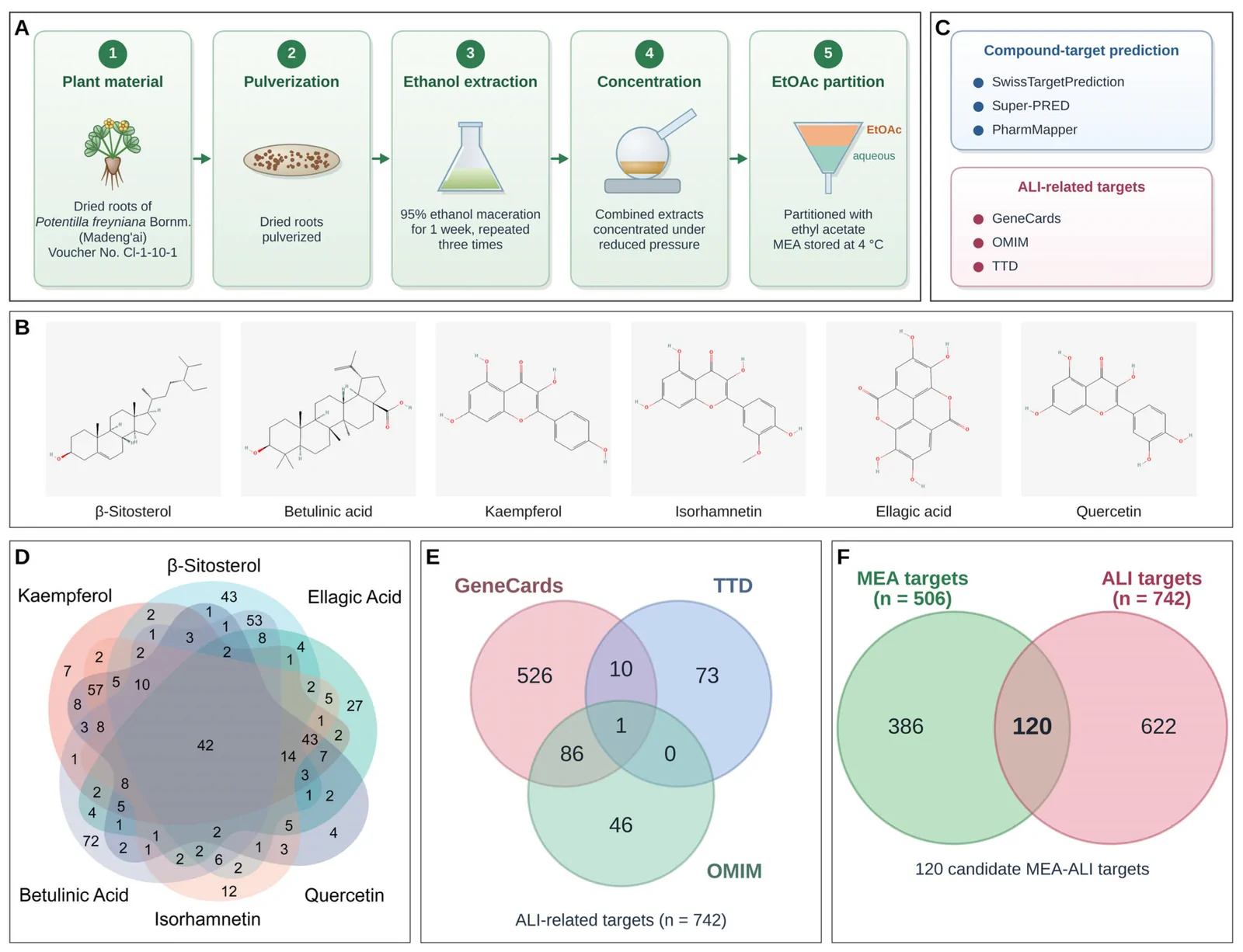
Extract preparation, candidate compounds, and target acquisition. (**A**) Preparation of MEA from the dried roots of *Potentilla freyniana* Bornm. (**B**) Structures of the six candidate compounds meeting the TCMSP thresholds (OB ≥ 30% and DL ≥ 0.18). (**C**) Databases used for compound–target prediction (SwissTargetPrediction, Super-PRED, and PharmMapper) and ALI-related target collection (GeneCards, OMIM, and TTD). (**D**) Targets predicted for the candidate compounds. (**E**) ALI-related targets from the three disease databases. (**F**) Intersection yielding 120 candidate MEA-ALI targets. Distinct colors in (**C**–**F**) differentiate individual databases and target sets.

Six detected constituents met the Traditional Chinese Medicine Systems Pharmacology Database and Analysis Platform (TCMSP) screening thresholds of oral bioavailability (OB) ≥ 30% and drug-likeness (DL) ≥ 0.18, comprising two reference-standard-confirmed compounds (ellagic acid and quercetin) and four compounds tentatively annotated by MS/MS (β-sitosterol, betulinic acid, kaempferol, and isorhamnetin) (Figure 2B; Table 2). Target prediction yielded 506 compound-related targets, while GeneCards, Online Mendelian Inheritance in Man (OMIM), and Therapeutic Target Database (TTD) yielded 742 ALI-related targets (Figure 2C–E). The intersection contained 120 candidate MEA-ALI targets (Figure 2F), which were used for exploratory network and enrichment analyses.

### 2.3. PPI Network and Enrichment of the 120 Candidate Targets

**Figure 3:**
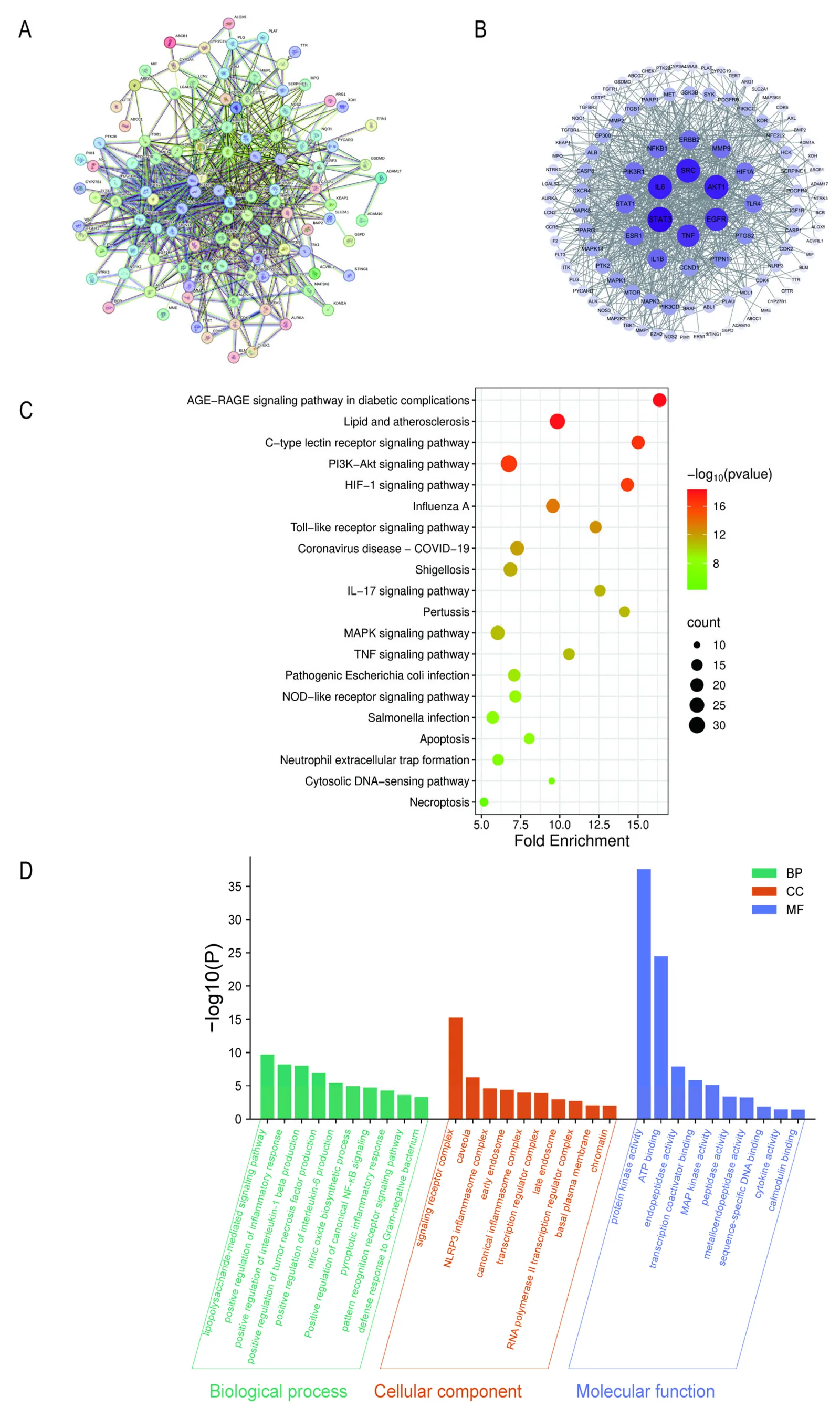
PPI network and functional enrichment of the 120 candidate MEA-ALI targets. (**A**) STRING network (interaction score > 0.7). (**B**) Cytoscape visualization; node size and color reflect degree. (**C**) KEGG pathway enrichment; dot size indicates gene count and color indicates significance. (**D**) GO enrichment of biological process (BP), cellular component (CC), and molecular function (MF) terms.

The 120 candidate MEA-ALI targets formed a densely connected STRING network at an interaction score greater than 0.7 (Figure 3A,B). Kyoto Encyclopedia of Genes and Genomes (KEGG) enrichment highlighted PI3K-Akt, HIF-1, Toll-like receptor, IL-17, MAPK, TNF, and NOD-like receptor signaling, together with pathways related to regulated cell death (Figure 3C); the inclusion of NLRP3 and CASP1 among the candidate targets guided our subsequent experimental evaluation of NLRP3–GSDMD pathway markers. Enriched Gene Ontology (GO) terms included lipopolysaccharide-mediated signaling, inflammatory and interleukin responses, canonical NF-κB signaling, kinase activity, and transcription-factor binding (Figure 3D).

### 2.4. Hub Targets Involved in Inflammatory Signaling

**Figure 4:**
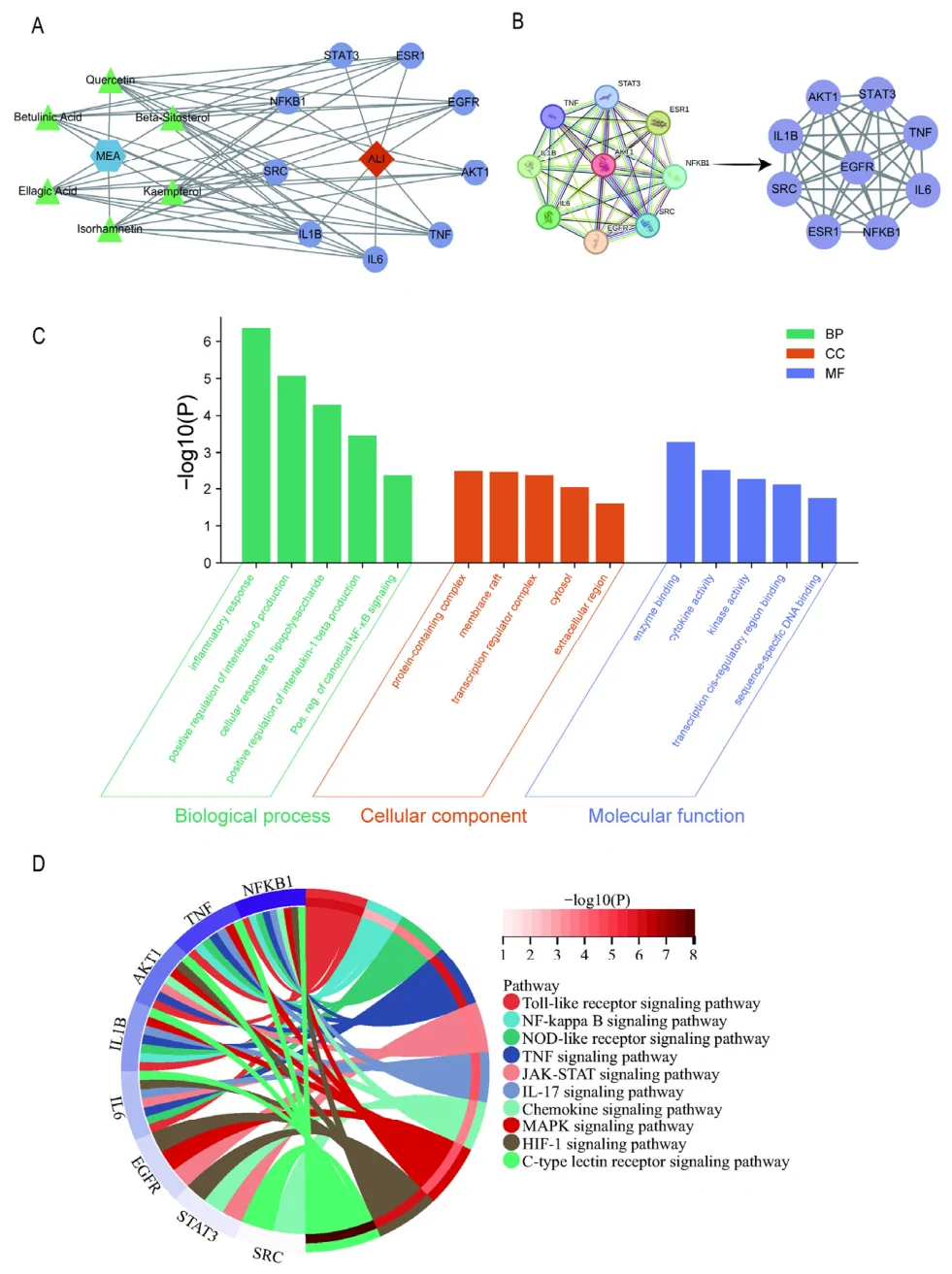
Hub-target screening and pathway interpretation. (**A**) Compound–target–disease network linking MEA, the six candidate compounds, ALI, and the nine hub targets. (**B**) Hub-target screening by maximal clique centrality, degree, closeness, and betweenness in cytoHubba; the intersection identified AKT1, EGFR, ESR1, IL1B, IL6, NFKB1, SRC, STAT3, and TNF. (**C**) GO enrichment of the nine hub targets. (**D**) Chord diagram linking hub targets to inflammation-related KEGG pathways.

Ranking by maximal clique centrality, degree, closeness, and betweenness identified nine overlapping hub targets: AKT1, EGFR, ESR1, IL1B, IL6, NFKB1, SRC, STAT3, and TNF. Their relationships with MEA and the six candidate compounds are shown in the compound–target–disease network (Figure 4A), and the topological screening is shown in Figure 4B. GO and KEGG analyses linked these predicted hub targets to LPS-responsive inflammatory signaling, cytokine production, and the NF-κB, Toll-like receptor, TNF, JAK-STAT, IL-17, MAPK, HIF-1, and NOD-like receptor pathways (Figure 4C,D). Functional descriptions of the nine targets are provided in Table 3.

### 2.5. Docking of Candidate Compounds with Inflammation-Related Targets

**Figure 5:**
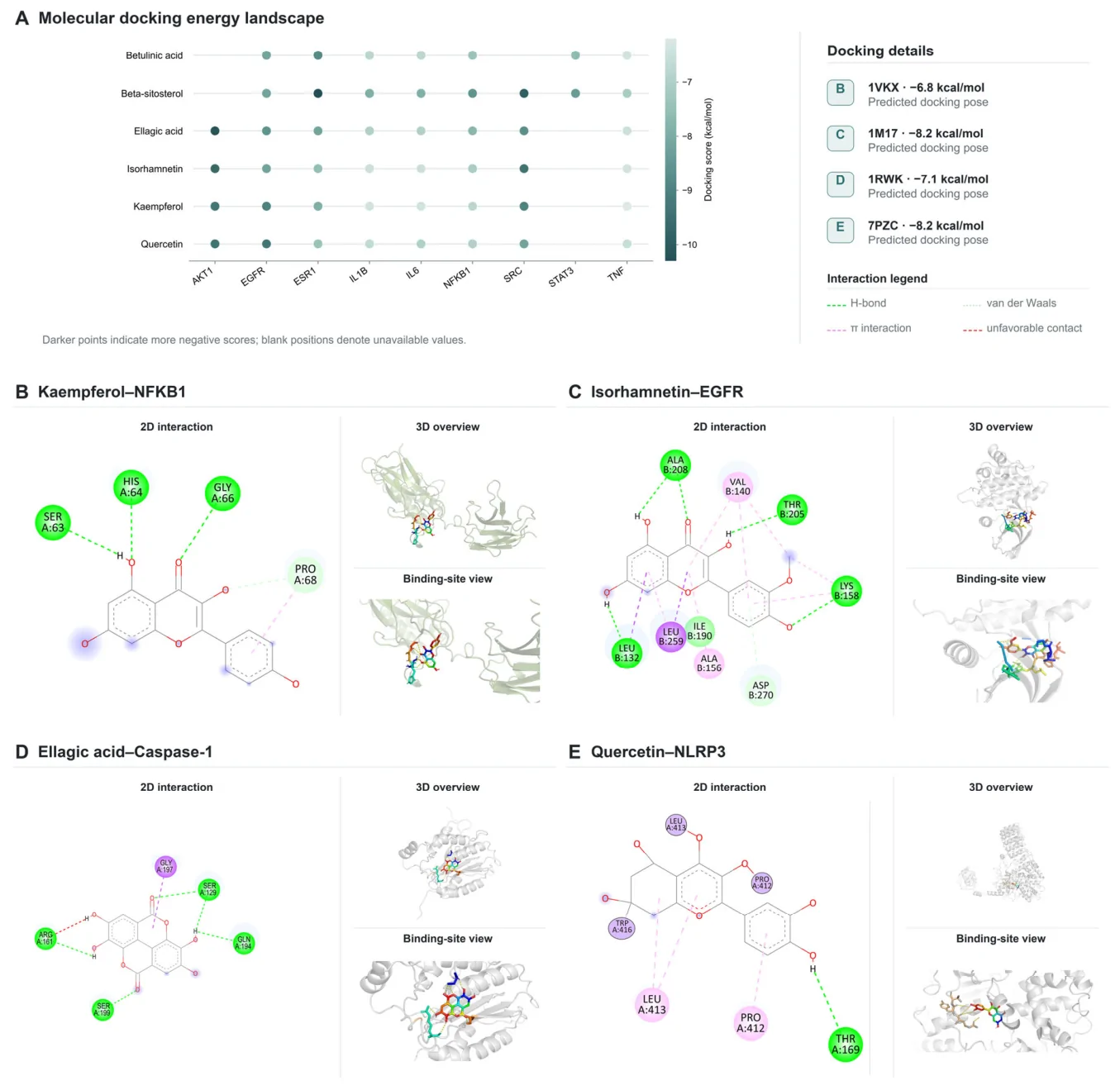
Molecular docking of candidate MEA compounds. (**A**) Dot map of AutoDock Vina binding energies (kcal/mol) for the six candidate compounds and the selected targets. (**B**–**E**) Two-dimensional interaction diagrams, three-dimensional overviews, and binding-pocket views for kaempferol-NFKB1 (PDB 1VKX) (**B**), isorhamnetin-EGFR (PDB 1M17) (**C**), ellagic acid-caspase-1 (PDB 1RWK) (**D**), and quercetin-NLRP3 (PDB 7PZC) (**E**). Dashed lines indicate hydrogen bonds and other non-covalent contacts. In (**A**), dot color and size reflect the predicted binding energy (kcal/mol); in (**B**–**E**), distinct colors distinguish the protein receptor, ligand, and interacting amino acid residues.

Predicted AutoDock Vina binding energies for the six candidate compounds across the screened targets are shown in Figure 5A. Four representative complexes were examined in detail to illustrate potential binding modes for the network-derived hubs NFKB1 and EGFR and the inflammasome-related proteins caspase-1 and NLRP3. Their predicted binding energies ranged from −6.8 to −8.2 kcal/mol: −6.8 kcal/mol for kaempferol-NFKB1, −8.2 kcal/mol for isorhamnetin-EGFR, −7.1 kcal/mol for ellagic acid-caspase-1, and −8.2 kcal/mol for quercetin-NLRP3. Predicted NFKB1 pocket contacts included Ser63, His64, Gly66, and Pro68. EGFR contacts included Leu132, Val140, Ala156, Lys158, Ile190, Thr205, Ala208, Leu259, and Asp270. Caspase-1 contacts included Ser129, Arg161, Gln194, Gly197, and Ser199, while NLRP3 contacts included Thr169, Pro412, Leu413, and Trp416 (Figure 5B–E).

### 2.6. Selection of Non-Cytotoxic MEA Concentrations

**Figure 6:**
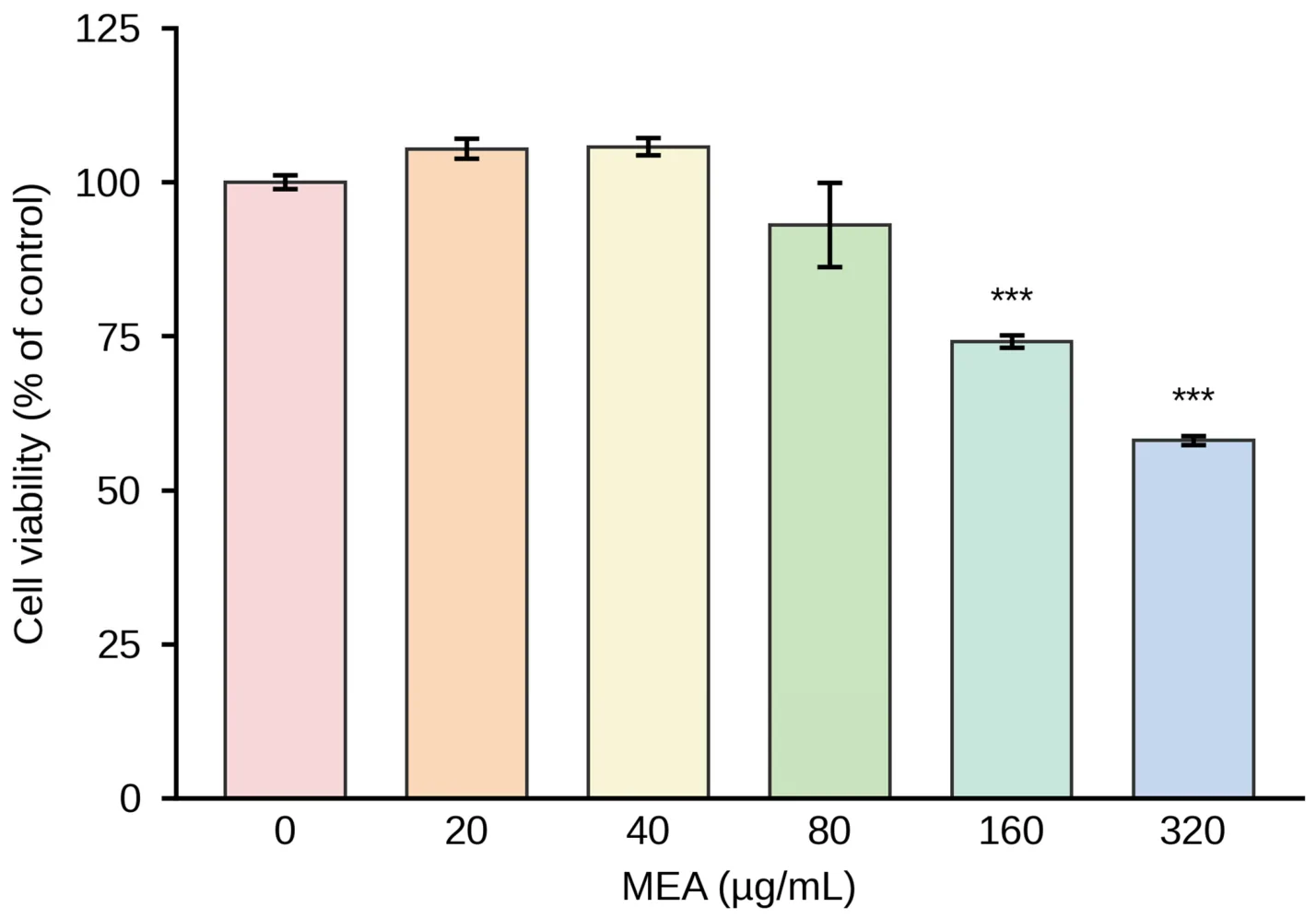
Effect of MEA on MH-S cell viability. Cells were treated with MEA (0–320 μg/mL) for 24 h, and viability was measured by Cell Counting Kit-8 (CCK-8) assay. Data are mean ± SD of *n* = 3 independent biological experiments; one-way ANOVA with Tukey’s test. *** *p* < 0.001 versus 0 μg/mL.

After 24 h of incubation, MH-S cell viability relative to the untreated control (100.21 ± 1.22%; normalized to 100.00%) was 101.78 ± 4.09% at 20 μg/mL, 101.97 ± 4.09% at 40 μg/mL, and 100.01 ± 2.55% at 80 μg/mL (*p* > 0.05 for all three concentrations versus 0 μg/mL), whereas viability decreased significantly to 71.87 ± 3.09% at 160 μg/mL and 58.63 ± 2.17% at 320 μg/mL (*p* < 0.001 versus 0 μg/mL; Figure 6). Because mean viability remained at ~100% across 20 to 80 μg/mL, any effect on basal cell survival within this concentration range was both statistically non-significant and biologically negligible, and 20, 40, and 80 μg/mL were therefore selected for subsequent cell experiments.

### 2.7. MEA Reduces Inflammatory Mediator Release in LPS-Stimulated MH-S Cells

**Figure 7:**
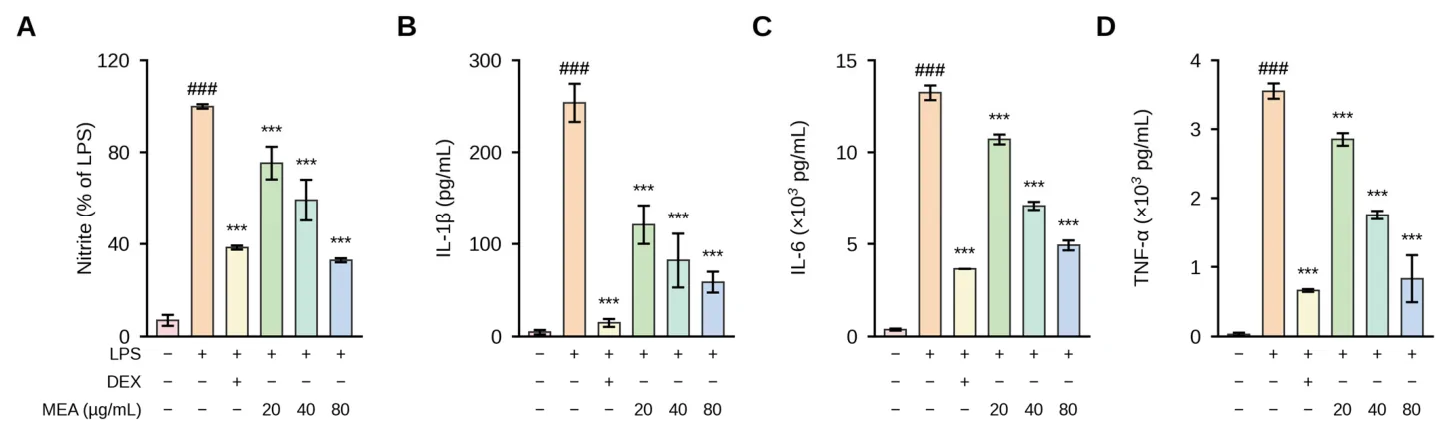
MEA reduces inflammatory mediator release in LPS-stimulated MH-S cells. Cells were pretreated with MEA (20, 40, or 80 μg/mL) or DEX (1 μg/mL) for 2 h and stimulated with LPS (1 μg/mL) for 24 h. (**A**) Nitrite (% of LPS), (**B**) IL-1β, (**C**) IL-6, and (**D**) TNF-α in culture supernatants. Data are mean ± SD of *n* = 3 independent biological experiments; one-way ANOVA with Tukey’s test. ### *p* < 0.001 versus Control; *** *p* < 0.001 versus LPS. “+” and “−” indicate the presence and absence of the corresponding treatment, respectively.

LPS stimulation significantly increased nitrite accumulation and interleukin (IL)-1β, IL-6, and tumor necrosis factor (TNF)-α concentrations in MH-S cell supernatants compared with the Control group (*p* < 0.001). Two-hour pretreatment with MEA at 20, 40, and 80 μg/mL significantly reduced all four inflammatory mediators relative to the LPS group (*p* < 0.001), with numerically lower group means observed at 40 and 80 μg/mL than at 20 μg/mL (Figure 7A–D). Pretreatment with dexamethasone (DEX; 1 μg/mL) similarly decreased the levels of these mediators (*p* < 0.001).

### 2.8. MEA Reduces ROS-Related Oxidative Fluorescence in LPS-Stimulated MH-S Cells

**Figure 8:**
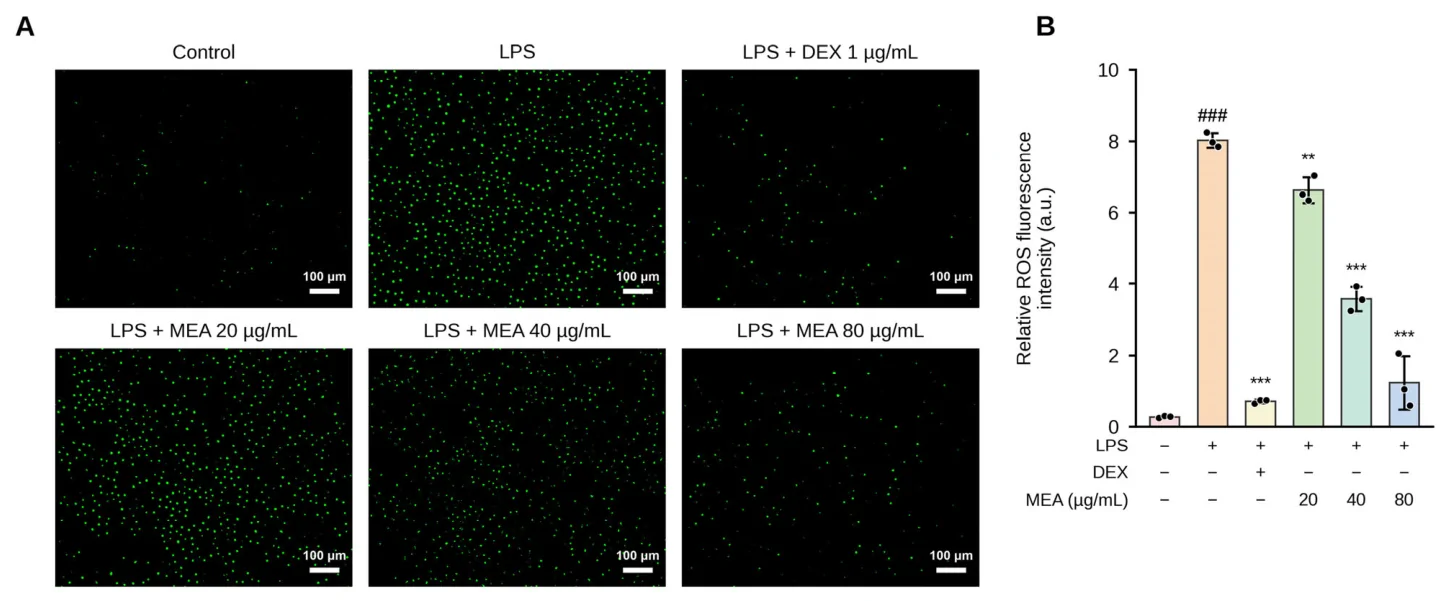
MEA reduces intracellular ROS-related oxidative fluorescence in LPS-stimulated MH-S cells. (**A**) Representative DCFH-DA fluorescence images. Scale bars: 100 μm. (**B**) Relative fluorescence intensity (a.u.), calculated as the mean green-channel intensity of each whole field. Data are mean ± SD of *n* = 3 independent biological experiments; dots show individual experiments. One-way ANOVA with Tukey’s test. ### *p* < 0.001 versus Control; ** *p* < 0.01, *** *p* < 0.001 versus LPS. “+” and “−” indicate the presence and absence of the corresponding treatment, respectively.

LPS stimulation markedly increased intracellular 2′,7′-dichlorodihydrofluorescein diacetate (DCFH-DA) fluorescence compared with Control cells (*p* < 0.001). Pretreatment with DEX and all three MEA concentrations significantly reduced this reactive oxygen species (ROS)-related oxidative fluorescence signal relative to the LPS group (*p* < 0.01 at 20 μg/mL; *p* < 0.001 at 40 and 80 μg/mL), with progressively lower group mean fluorescence intensity observed across 20, 40, and 80 μg/mL MEA (Figure 8A,B).

### 2.9. MEA Downregulates NLRP3–GSDMD Pathway Genes in MH-S Cells

**Figure 9:**
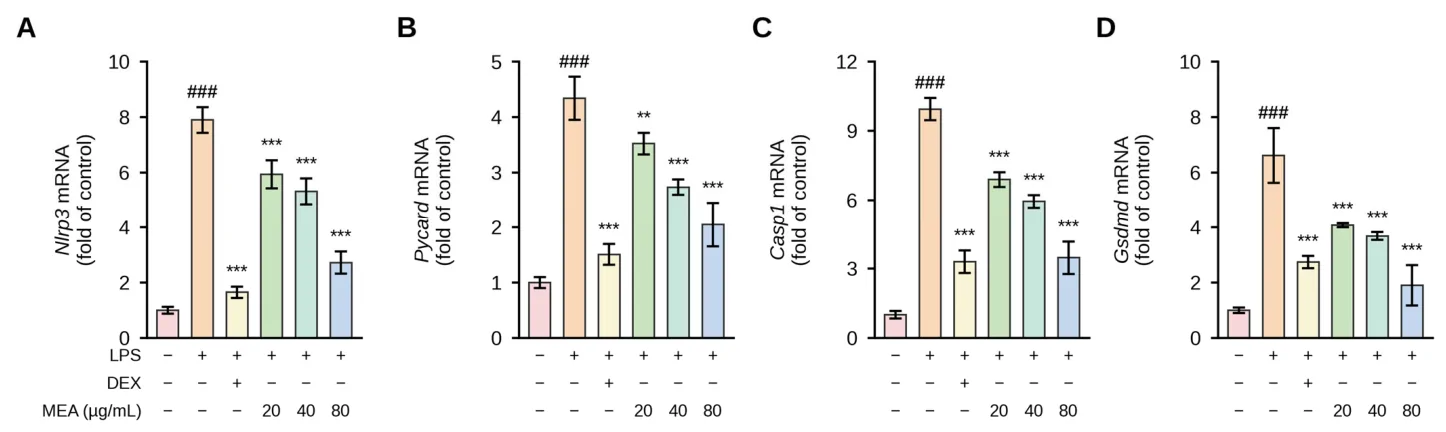
MEA decreases NLRP3–GSDMD pathway-related gene expression in LPS-stimulated MH-S cells. *Nlrp3* (**A**), *Pycard* (**B**), *Casp1* (**C**), and *Gsdmd* (**D**) mRNA levels were measured by reverse transcription quantitative polymerase chain reaction (RT-qPCR), normalized to *Actb*, and expressed relative to Control. Data are mean ± SD of *n* = 3 independent biological experiments; one-way ANOVA with Tukey’s test. ### *p* < 0.001 versus Control; ** *p* < 0.01, *** *p* < 0.001 versus LPS. “+” and “−” indicate the presence and absence of the corresponding treatment, respectively.

LPS stimulation significantly increased *Nlrp3*, *Pycard*, *Casp1*, and *Gsdmd* mRNA expression in MH-S cells compared with the Control group (*p* < 0.001). Pretreatment with DEX and MEA (20, 40, and 80 μg/mL) significantly reduced these transcript levels relative to the LPS group (*p* < 0.01 or *p* < 0.001), with progressively lower mean transcript levels observed from 20 to 80 μg/mL MEA (Figure 9A–D).

### 2.10. MEA Attenuates Lung Injury, Edema, and Circulating Cytokines in ALI Mice

**Figure 10:**
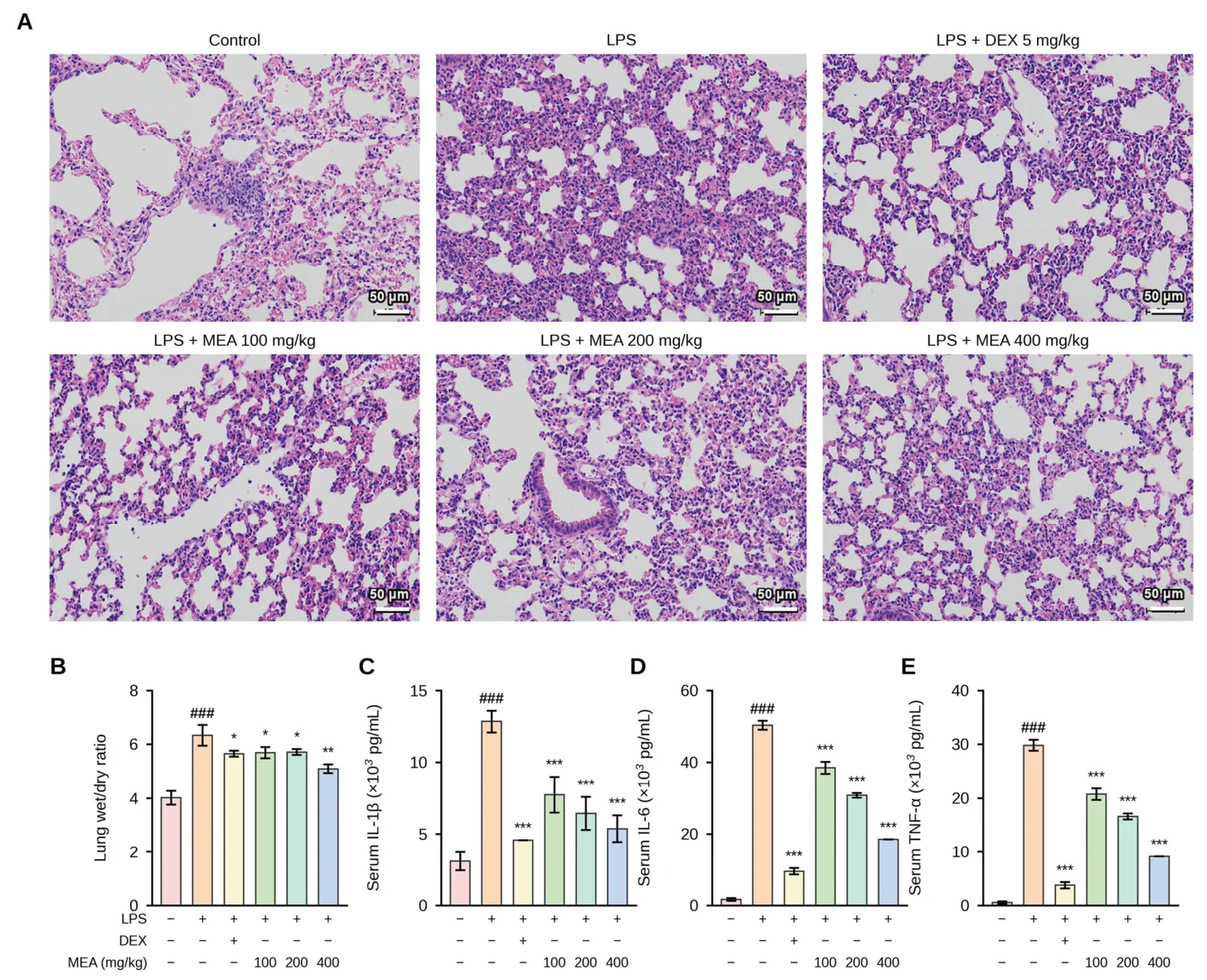
MEA attenuates lung injury, edema, and serum cytokines in LPS-induced ALI mice. Mice received MEA (100, 200, or 400 mg/kg) or DEX (5 mg/kg) by oral gavage once daily for 7 days, followed by LPS (20 mg/kg, i.p.) for 12 h. (**A**) Representative H&E-stained lung sections. Scale bars: 50 μm. (**B**) Lung wet/dry weight ratio. (**C**–**E**) Serum IL-1β (**C**), IL-6 (**D**), and TNF-α (**E**). Data are mean ± SD, *n* = 10 mice per group; one-way ANOVA with Tukey’s test. ### *p* < 0.001 versus Control; * *p* < 0.05, ** *p* < 0.01, *** *p* < 0.001 versus LPS. “+” and “−” indicate the presence and absence of the corresponding treatment, respectively.

Qualitative histological examination of hematoxylin and eosin (H&E)-stained lung sections showed pronounced alveolar septal thickening, inflammatory-cell accumulation, and disruption of normal alveolar architecture in the LPS group, whereas these morphological alterations appeared less extensive in mice receiving seven-day oral pretreatment with DEX or MEA (Figure 10A). Quantitatively, LPS challenge significantly increased the lung wet/dry weight ratio compared with the Control group (*p* < 0.001), whereas oral pretreatment with DEX or MEA (100, 200, and 400 mg/kg) significantly reduced this index of pulmonary edema (*p* < 0.05 or *p* < 0.01 versus LPS; Figure 10B). Serum IL-1β, IL-6, and TNF-α concentrations were also markedly elevated 12 h after LPS challenge (*p* < 0.001) and were significantly lower in mice pretreated with DEX or MEA across the 100 to 400 mg/kg dose range (*p* < 0.001 versus LPS; Figure 10C–E).

### 2.11. MEA Decreases Lung NLRP3–GSDMD Pathway Proteins and Transcripts

**Figure 11:**
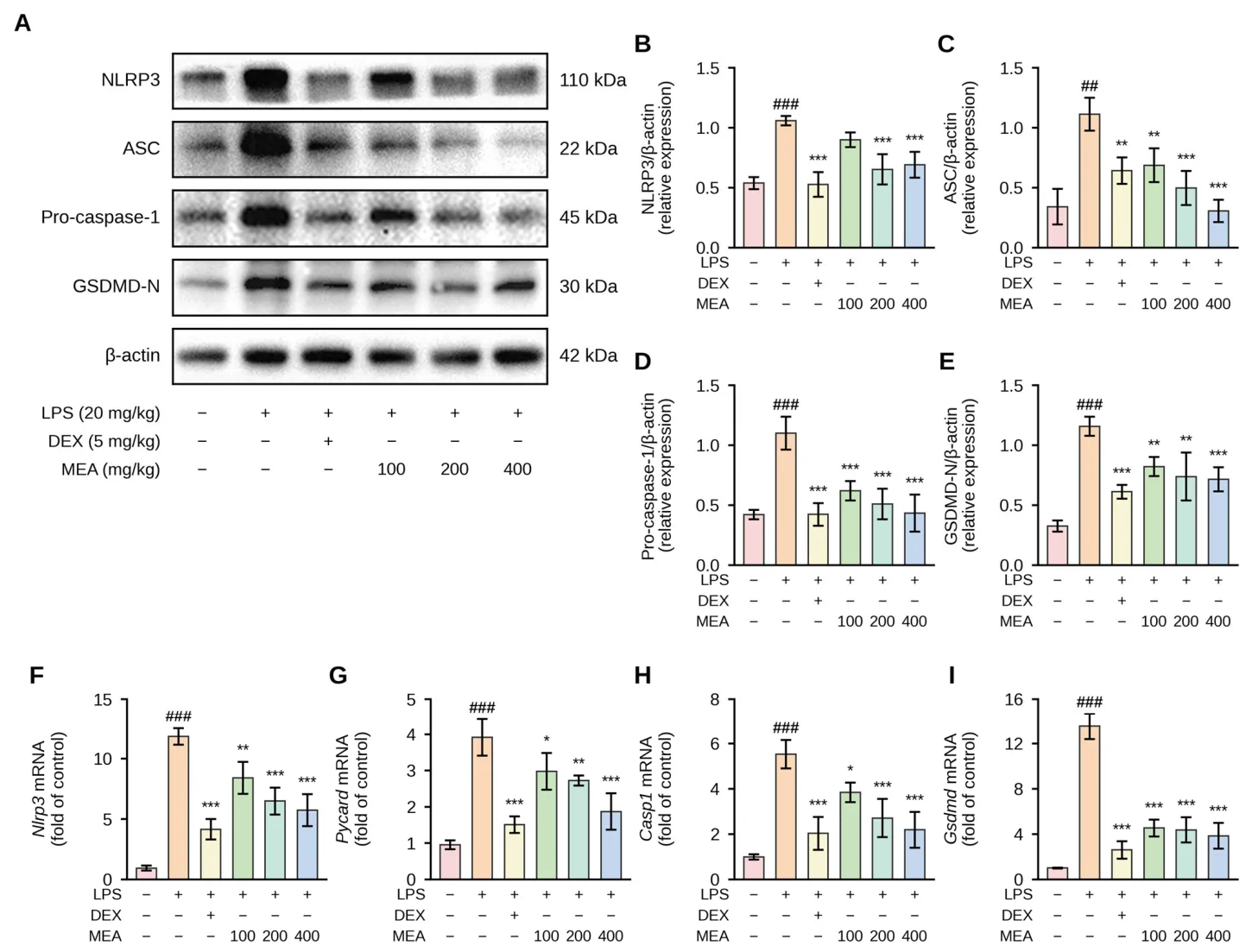
MEA decreases lung NLRP3–GSDMD pathway-related protein and gene expression in LPS-induced ALI mice. (**A**) Representative Western blots of NLRP3, ASC, pro-caspase-1 (~45 kDa), GSDMD-N (~30 kDa), and β-actin in lung tissue. (**B**–**E**) Densitometry normalized to β-actin (*n* = 3 randomly selected mice per group). (**F**–**I**) *Nlrp3*, *Pycard*, *Casp1*, and *Gsdmd* mRNA normalized to *Actb* (*n* = 10 mice per group). Data are mean ± SD; one-way ANOVA with Tukey’s test. ## *p* < 0.01, ### *p* < 0.001 versus Control; * *p* < 0.05, ** *p* < 0.01, *** *p* < 0.001 versus LPS. “+” and “−” indicate the presence and absence of the corresponding treatment, respectively.

LPS challenge significantly increased lung protein levels of NLRP3, ASC, pro-caspase-1 (~45 kDa), and the ~30 kDa GSDMD-N cleavage fragment compared with the Control group (*p* < 0.01 or *p* < 0.001), whereas seven-day oral pretreatment with DEX or MEA (100, 200, and 400 mg/kg) significantly reduced the levels of these four protein markers relative to the LPS group (Figure 11A–E). Concordantly, lung *Nlrp3*, *Pycard*, *Casp1*, and *Gsdmd* mRNA levels were significantly elevated after LPS challenge (*p* < 0.001, *n* = 10 mice per group) and were significantly decreased following pretreatment with DEX or MEA (Figure 11F–I).

## 3. Discussion

**Figure 12:**
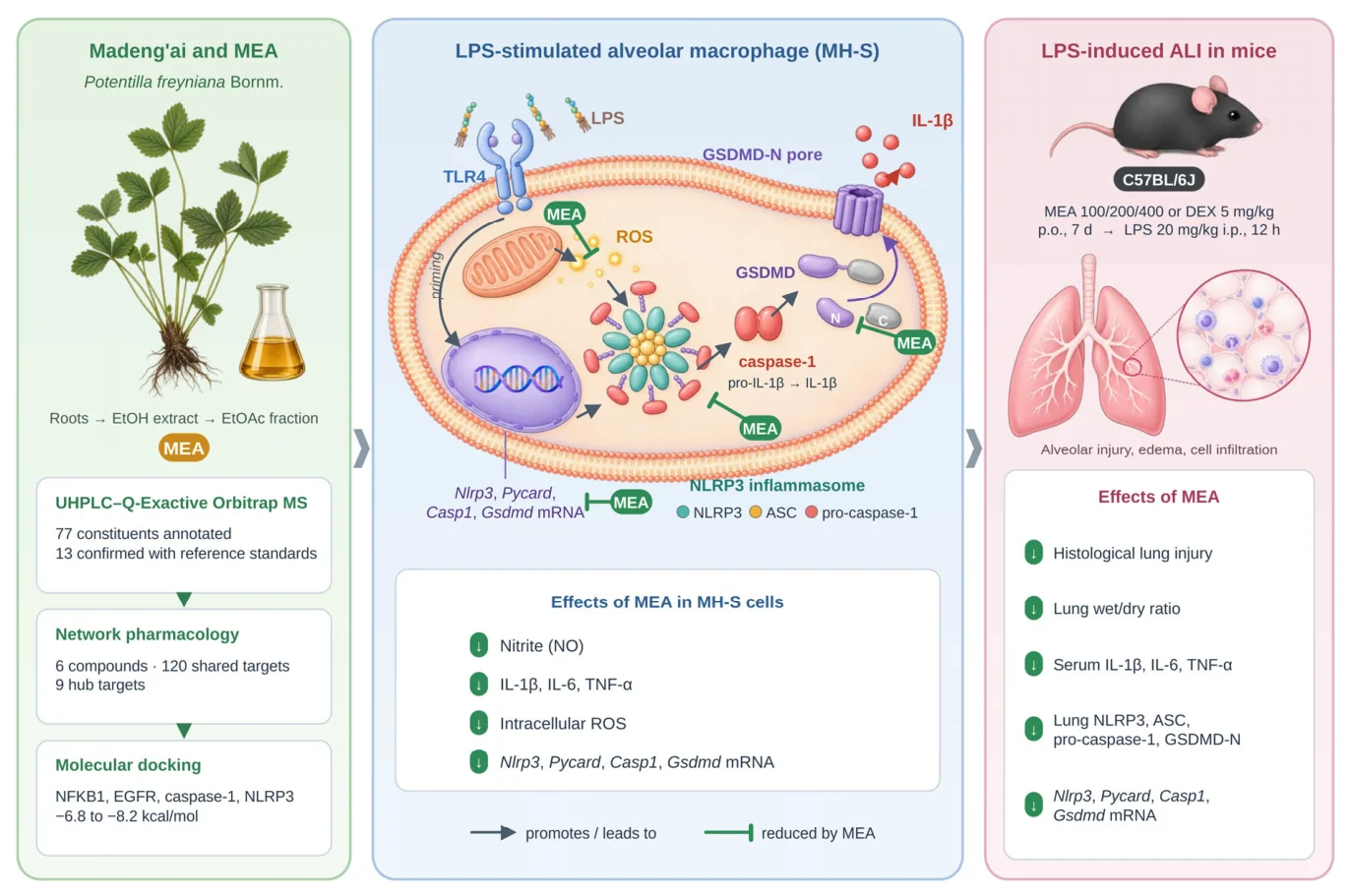
Summary of chemical profiling and proposed associative mechanism of MEA in LPS-induced acute lung injury. The schematic links MEA constituents and computationally predicted targets with reduced macrophage inflammatory mediator release and ROS-related oxidative fluorescence, decreased lung expression of NLRP3, ASC, pro-caspase-1, and the ~30 kDa GSDMD-N fragment, and alleviated lung injury following prophylactic administration. Blunt-ended lines indicate associative downregulation of pathway markers.

Seven-day oral pretreatment with MEA attenuated LPS-induced pulmonary edema, histological lung injury, and systemic cytokine elevation in mice while suppressing inflammatory mediator release in LPS-stimulated MH-S alveolar macrophages. These protective effects were accompanied by lower intracellular DCFH-DA oxidative fluorescence and reduced expression of NLRP3–GSDMD pathway-associated transcripts and proteins (Figure 12). These findings both corroborate and extend our recently published multi-omics study of the ethyl acetate fraction of Madeng’ai in murine ALI [15]. While the earlier study used an independent animal cohort to profile global pulmonary transcriptomic and metabolomic remodeling and relied on tentative untargeted MS annotation [15], the present independent dataset advances those observations by authenticating 13 marker constituents of MEA with reference standards alongside 64 MS/MS-annotated peaks (Table 1; Supplementary Table S1), demonstrating direct anti-inflammatory and redox-modulating effects in cultured MH-S alveolar macrophages, and showing that MEA downregulates *Nlrp3*, *Pycard*, *Casp1*, and *Gsdmd* mRNA as well as NLRP3, ASC, pro-caspase-1, and ~30 kDa GSDMD-N protein levels in lung tissue.

Given the chemical complexity of MEA, network pharmacology was used to prioritize candidate inflammatory pathways. The 120 overlapping targets were enriched in LPS-responsive signaling, and the nine topological hub targets included IL1B, IL6, TNF, and NFKB1—aligning with the pro-inflammatory cytokines reduced in our macrophage and mouse experiments and with NF-κB-dependent transcriptional priming of the inflammasome. Because database target predictions and fixed TCMSP thresholds (OB ≥ 30% and DL ≥ 0.18) favor small orally absorbable molecules and four of the six filtered candidates (kaempferol, isorhamnetin, betulinic acid, and β-sitosterol) were tentatively annotated by MS/MS, the network and KEGG enrichment results (including untested pathways such as PI3K-Akt, HIF-1, MAPK, and IL-17) serve as hypothesis-generating prioritizations.

Reference-standard authentication confirmed that MEA contains both polyphenols/flavonoids (gallic acid, procyanidin B1, catechin, taxifolin, epicatechin gallate, ellagic acid, phlorizin, quercetin, naringenin, and phloretin) and ursane-type pentacyclic triterpenoids (rosamultin, pomolic acid, and ursolic acid). Prior studies have shown that quercetin, ellagic acid, and catechins suppress macrophage oxidative stress and cytokine production [16,17,18], while ursolic acid and pomolic acid inhibit NF-κB activation, attenuate reactive oxygen species generation, and reduce IL-1β release in experimental inflammation [12,13,19,20]. Because our UHPLC-Q-Exactive Orbitrap MS analysis was qualitative and biological assays were conducted with the whole MEA fraction, purified-monomer and combination assays will be needed to determine the individual potency and potential interactions of these constituents. In MH-S macrophages, MEA reduced DCFH-DA oxidative fluorescence [21] alongside inflammasome-related transcripts, consistent with coordinated attenuation of macrophage oxidative stress and inflammatory signaling [22,23].

Molecular docking examined theoretical binding complementarity between selected candidate constituents and the network hubs NFKB1 and EGFR as well as the inflammasome-related proteins NLRP3 and caspase-1. The predicted binding energies (−6.8 to −8.2 kcal/mol) and hydrogen-bonding contacts within static crystal structures provide structural hypotheses for how polyphenols and flavonoids in MEA may interact with inflammatory signaling proteins [24], offering candidate compound–target pairs for future biophysical binding and target-engagement studies.

In canonical inflammasome signaling, caspase-1 activation cleaves GSDMD to release the N-terminal fragment GSDMD-N, which forms plasma membrane pores and drives inflammatory cell injury [5,6]. Here, prophylactic MEA administration reduced lung NLRP3, ASC, pro-caspase-1 (~45 kDa), and ~30 kDa GSDMD-N protein levels (assessed by Western blot in *n* = 3 randomly selected mice per group) alongside *Nlrp3*, *Pycard*, *Casp1*, and *Gsdmd* mRNA in macrophages and lung tissue (*n* = 10 mice per group), accompanying improvements in pulmonary edema and systemic cytokines [7,8]. These coordinated reductions indicate that MEA dampens NLRP3–GSDMD pathway expression and upstream inflammatory priming in LPS-challenged lungs; direct functional assays—including cleaved caspase-1 (p20) activity, ASC oligomerization, lactate dehydrogenase release, propidium iodide uptake, and selective NLRP3 inhibition or knockdown—will be required to determine whether MEA directly blocks inflammasome assembly and pyroptotic pore formation.

Several additional study boundaries merit consideration. First, although a single chemically fingerprinted batch of MEA (extraction yield 4.80%, *w*/*w*) was used across all experiments, multi-batch quantitative standardization of marker constituents and in vivo pharmacokinetic profiling [25] remain to be established. Second, acute lung injury was induced in male C57BL/6J mice by systemic intraperitoneal LPS injection (20 mg/kg for 12 h) following 7-day oral MEA pretreatment, which models prophylactic protection against endotoxemia-associated indirect lung injury; future studies should evaluate both sexes and post-challenge therapeutic administration in direct intrapulmonary ARDS models [26]. Third, pulmonary H&E histology was evaluated qualitatively alongside quantitative lung wet/dry weight ratios and serum cytokine measurements, and incorporating blinded histological scoring will further strengthen pathological assessment.

## 4. Materials and Methods

### 4.1. Plant Material, Extract Preparation, and Reagents

Madeng’ai roots (*Potentilla freyniana* Bornm.) were provided by the Hunan Provincial Key Laboratory of Dong Medicine and authenticated by Prof. Zaiqi Zhang (Hunan University of Medicine). A voucher specimen (No. Cl-1-10-1) was deposited at the Hunan Provincial Key Laboratory of Dong Medicine (Huaihua, China). Dried roots (5.0 kg) were pulverized and macerated in 95% ethanol at a solid-to-liquid ratio of 1:10 (*w*/*v*) at room temperature for 7 days, and extraction was repeated three times. The combined ethanolic filtrates were concentrated under reduced pressure to remove ethanol, suspended in distilled water at a ratio of 1:1.5 (*w*/*v*), and successively partitioned three times with an equal volume of ethyl acetate (1:1, *v*/*v*). The combined ethyl acetate layers were concentrated under reduced pressure and dried to yield 240 g of the ethyl acetate fraction of the ethanolic root extract of Madeng’ai (referred to as MEA; extraction yield 4.80%, *w*/*w*, relative to dried crude root). A single homogenized batch of MEA, chemically fingerprinted by UHPLC-Q-Exactive Orbitrap MS, was stored in sealed amber vials at 4 °C in the dark until use across all experiments. Dexamethasone (DEX; ST1254) was purchased from Beyotime Biotechnology (Shanghai, China), and LPS (L2880) was obtained from Sigma-Aldrich (St. Louis, MO, USA).

HPLC-grade acetonitrile and methanol were purchased from Merck KGaA (Rahway, NJ, USA). Purified water was obtained from Watsons (Changsha, China), and formic acid was provided by Fisher Scientific (Fair Lawn, NJ, USA). Reference standards were sourced as follows: gallic acid and epicatechin gallate from Chengdu Zhibiao Chemical Pure Biotechnology Co., Ltd. (Chengdu, China); catechin and quercetin from Shanghai Yuanye Bio-Technology Co., Ltd. (Shanghai, China); taxifolin and ellagic acid from Chengdu Alfa Biotechnology Co., Ltd. (Chengdu, China); procyanidin B1 from Sichuan Vicki Biotechnology Co., Ltd. (Chengdu, China); phlorizin from Chengdu Lemeitian Pharmaceutical Technology Co., Ltd. (Chengdu, China); naringenin from Chengdu Herbpurify Co., Ltd. (Chengdu, China); phloretin from Nanjing Spring & Autumn Biological Engineering Co., Ltd. (Nanjing, China); and ursolic acid from the National Institutes for Food and Drug Control (Beijing, China). Rosamultin and pomolic acid were isolated in our laboratory. The purity of all reference standards was greater than 98%. All other solvents were of analytical grade.

### 4.2. UHPLC-Q-Exactive Orbitrap MS Analysis

UHPLC analysis was performed using an Ultimate 3000 system (Thermo Fisher Scientific, Waltham, MA, USA) equipped with a Thermo Scientific Syncronis aQ column (100 mm × 2.1 mm, 1.7 μm) maintained at 40 °C. The mobile phases were 0.1% formic acid in water (A) and acetonitrile (B), delivered at 0.3 mL/min. The gradient was 95% A at 0–3 min; 90% A at 3–8 min; 90–75% A at 8–12 min; 75–70% A at 12–16 min; 70–45% A at 16–21 min; 45–5% A at 21–25 min; 5–95% A at 25–26 min; and 95% A at 26–30 min. The injection volume was 2 μL.

Mass spectrometry was performed on a Q Exactive mass spectrometer (Thermo Fisher Scientific, Bremen, Germany) with a heated electrospray ionization source. Data were acquired separately in positive and negative ion modes over *m/z* 100–1500. The source parameters were sheath gas (N_2_), 30 arbitrary units; auxiliary gas (N_2_), 10 arbitrary units; capillary temperature, 320 °C; source heater temperature, 350 °C; and spray voltage magnitude, 3.0 kV. MS1 spectra were acquired in full-scan mode at a resolution of 35,000. MS2 spectra were obtained by data-dependent acquisition of the three most intense precursor ions with a normalized collision energy of 30%. Compounds were assigned from accurate mass, MS/MS fragmentation, and, where available, comparison of retention time and spectra with reference standards.

### 4.3. Candidate Compound Screening and Target Prediction

Because MEA was administered by oral gavage in the in vivo study, the 77 detected constituents were queried in the TCMSP platform (version 2.3) [27] and filtered using the conventional pharmacokinetic screening criteria of predicted oral bioavailability (OB) ≥ 30% (reflecting predicted gastrointestinal absorption and first-pass metabolic stability) and drug-likeness (DL) ≥ 0.18 (based on the average Tanimoto similarity of small-molecule drugs in DrugBank) to prioritize candidate orally absorbable small molecules for exploratory network construction. Six compounds met these thresholds, comprising two reference-standard-confirmed constituents (ellagic acid and quercetin) and four constituents tentatively assigned from accurate mass and MS/MS fragmentation (kaempferol, isorhamnetin, betulinic acid, and β-sitosterol). Because fixed OB and DL cutoffs may exclude bioactive glycosides, tannins, or triterpenoids that act via intestinal metabolites or local signaling, this filtering was used for candidate prioritization in network construction. Compound-related targets were predicted using SwissTargetPrediction (2019 version), Super-PRED (version 3.0), and PharmMapper (2017 version) [28,29,30]. Target names were standardized to official human gene symbols using UniProt (release 2023_05) [31], and duplicates were removed. ALI-related targets were collected from GeneCards (version 5.18), OMIM (2023 release), and the Therapeutic Target Database (TTD, 2024 update) using “acute lung injury” as the search term [32,33,34]. The intersection of compound-related and ALI-related targets was defined as the candidate MEA-ALI target set.

### 4.4. PPI Network Construction, Hub-Target Screening, and Enrichment Analysis

Candidate MEA-ALI targets were submitted to STRING (version 12.0) for protein–protein interaction (PPI) analysis, with the organism set to *Homo sapiens* and the minimum required interaction score set to greater than 0.7 [35]. The interaction file was imported into Cytoscape (version 3.9.1) for visualization and network analysis [36]. Gene Ontology (GO) enrichment analysis was performed for biological process, cellular component, and molecular function categories, and Kyoto Encyclopedia of Genes and Genomes (KEGG) pathway enrichment analysis was used to identify disease-relevant signaling pathways. The 120 candidate targets were ranked with the cytoHubba plug-in (version 0.1) using maximal clique centrality, degree, closeness, and betweenness [37]. The top 10 targets from each algorithm were extracted, and their intersection was defined as the core target set. A compound–target–disease network was constructed to show the relationships among MEA, the candidate compounds, ALI, and the core targets, which were further subjected to GO/KEGG enrichment analyses.

### 4.5. Molecular Docking

Exploratory molecular docking was performed to evaluate theoretical binding complementarity between candidate MEA constituents and inflammation-related proteins. While binding energies were calculated across the six TCMSP-screened candidate compounds and the core targets, four representative complexes were selected for detailed structural visualization to cover two upstream network hubs (NFKB1 and EGFR) and two focal inflammasome-related proteins (caspase-1 and NLRP3), paired with the two reference-standard-confirmed candidate polyphenols (ellagic acid for caspase-1 and quercetin for NLRP3) and two candidate flavonoids (kaempferol for NFKB1 and isorhamnetin for EGFR). Crystal structures were obtained from the RCSB Protein Data Bank (NFKB1, PDB ID 1VKX; EGFR, 1M17; caspase-1, 1RWK; NLRP3, 7PZC). Using AutoDockTools (version 1.5.7; The Scripps Research Institute, La Jolla, CA, USA), crystallographic water molecules and non-interacting heteroatoms were removed, polar hydrogen atoms were added, and Kollman charges were assigned to the receptors; three-dimensional ligand structures from PubChem were energy-minimized and assigned Gasteiger partial charges with rotatable bonds defined. Docking grid boxes were centered on the active or ligand-binding pockets as follows: NFKB1 (1VKX), center (x = −19.5, y = 21.1, z = 99.5) Å, size 22 × 22 × 22 Å; EGFR (1M17), center (x = 22.0, y = 0.3, z = 52.8) Å, size 24 × 20 × 20 Å; caspase-1 (1RWK), center (x = 34.8, y = 61.0, z = 2.5) Å, size 24 × 24 × 24 Å; and NLRP3 (7PZC), center (x = 192.9, y = 204.7, z = 119.7) Å, size 24 × 24 × 24 Å. Docking was executed in AutoDock Vina (version 1.2.0; exhaustiveness = 16) [38] after verifying pocket coordinates against native co-crystallized ligand positions, and predicted binding energies were reported in kcal/mol. Two-dimensional interaction maps and three-dimensional docking-site views were generated using Discovery Studio Visualizer (version 2021; Dassault Systèmes, San Diego, CA, USA) and PyMOL (version 2.5; Schrödinger, LLC, New York, NY, USA) for representative complexes.

### 4.6. Cell Culture and Cytotoxicity Assay

MH-S cells were obtained from the China Center for Type Culture Collection (Wuhan, China) and maintained in RPMI 1640 medium containing 10% fetal bovine serum and 1% penicillin-streptomycin at 37 °C in 5% CO2. Cytotoxicity was assessed using a Cell Counting Kit-8 (CCK-8) assay (Beyotime Biotechnology, Shanghai, China). Cells were seeded in 96-well plates at 7 × 103 cells per well and incubated overnight, followed by treatment with MEA (0, 20, 40, 80, 160, or 320 μg/mL; final DMSO < 0.1% *v*/*v* across all wells) for 24 h. CCK-8 reagent was added for 1.5 h in the dark, and absorbance was measured at 450 nm. Technical replicate wells were averaged within each experiment to yield one value per run, and three independent biological experiments (*n* = 3) were performed on different days using independent cell passages and freshly prepared working dilutions.

### 4.7. Cellular Inflammatory Model and Treatment

MH-S cells were assigned to Control, LPS, DEX, and MEA treatment groups. MEA was dissolved in sterile DMSO and diluted in complete RPMI 1640 medium to 20, 40, or 80 μg/mL, with a final DMSO concentration below 0.1% (*v*/*v*); Control and LPS groups received medium containing the equivalent concentration of DMSO vehicle (<0.1% *v*/*v*). DEX was used as a positive anti-inflammatory control at 1 μg/mL based on preliminary testing and established LPS-stimulated macrophage protocols [15]. Cells were seeded in 6-well plates at 5 × 105 cells per well and incubated overnight. MEA or DEX was added 2 h before stimulation with LPS (1 μg/mL) for 24 h. Supernatants were collected for ELISA and nitrite measurement, and cells were used for intracellular oxidative fluorescence imaging and RT-qPCR. Each cellular assay was performed in three independent biological experiments (*n* = 3) conducted on separate days using independent cell cultures and freshly prepared treatment solutions.

### 4.8. Intracellular ROS Measurement

After treatment, cells were incubated with 10 μM 2′,7′-dichlorodihydrofluorescein diacetate (DCFH-DA) at 37 °C for 30 min in the dark. Fluorescence images were acquired at excitation/emission wavelengths of 488/525 nm under identical acquisition settings for all groups. The mean green-channel intensity of each whole field was quantified in ImageJ (version 1.53t; National Institutes of Health, Bethesda, MD, USA) and expressed in arbitrary units. Each group contributed one value from each of three independent experiments. DCFH-DA fluorescence was used to assess intracellular ROS-related oxidation [21].

### 4.9. Nitrite Assay

Nitric oxide production was estimated from nitrite concentrations in MH-S cell supernatants using a Griess kit (Beyotime Biotechnology, Shanghai, China). Standards and samples were added to 96-well plates, followed by Griess reagents I and II. Absorbance was measured at 540 nm, and nitrite concentrations were calculated from the standard curve and expressed relative to the LPS group.

### 4.10. Animals and Experimental Design

Sixty male C57BL/6J mice (6–8 weeks old, 20 ± 2 g) were obtained from Hunan Slack Jingda Experimental Animal Co., Ltd. (Changsha, China) as an independent animal cohort distinct from our previously published multi-omics study [15]. Male mice were used to minimize estrous-cycle-associated hormonal variability in acute LPS-induced innate immune responses. Animals were housed at Hunan University of Medicine with standard chow and water available ad libitum under a 12 h light/dark cycle at 20–26 °C. The protocol was approved by the Medical Ethics Committee of Hunan University of Medicine (approval no. 2024(A01001); approved on 6 January 2024).

After five days of acclimation, mice were randomly assigned to six groups (*n* = 10 per group): Control, LPS, DEX (5 mg/kg), and MEA (100, 200, or 400 mg/kg). Control and LPS mice received an equivalent volume of 0.9% saline, whereas the other groups received prophylactic oral gavage of DEX or MEA once daily for 7 days. One hour after the final administration, systemic endotoxemia-associated ALI was induced by intraperitoneal injection of LPS (20 mg/kg), which reproducibly triggers acute pulmonary microvascular barrier dysfunction, edema, and leukocyte recruitment [7,15,26]; Control mice received an equal volume of sterile saline. Samples were collected 12 h after LPS challenge during the acute hyperinflammatory phase. The doses of DEX (5 mg/kg) and MEA (100, 200, and 400 mg/kg) and the 7-day pretreatment schedule were selected based on preliminary dose-ranging experiments and established murine ALI protocols [7,15].

### 4.11. Specimen Collection and Lung Wet/Dry Ratio

At 12 h after LPS challenge, mice were anesthetized by intraperitoneal injection of ketamine (80–100 mg/kg) and xylazine (10 mg/kg). Adequate anesthetic depth was confirmed by loss of the pedal withdrawal reflex. Blood was collected, and mice were then euthanized by cervical dislocation under deep anesthesia in accordance with the AVMA Guidelines for the Euthanasia of Animals (2020 Edition) [39]. Death was confirmed by the absence of spontaneous respiration, heartbeat, and reflexes. Serum was isolated by centrifugation at 3000 rpm for 30 min at 4 °C and stored at −80 °C. Lungs were excised immediately and allocated for histology, RNA extraction, protein analysis, and wet/dry measurement. For the wet/dry assay, fresh lung tissue was weighed, dried at 60 °C for 48 h to constant mass, and weighed again; the wet/dry ratio was calculated as wet mass divided by dry mass.

### 4.12. Hematoxylin and Eosin Staining

Paraformaldehyde-fixed lung tissues were dehydrated through graded ethanol, embedded in paraffin, and sectioned at 5 μm. Sections were stained with hematoxylin and eosin (H&E) using standard procedures and examined qualitatively under a ZEISS light microscope (Carl Zeiss, Oberkochen, Germany) to evaluate morphological alterations, including alveolar septal thickening and inflammatory cell infiltration.

### 4.13. ELISA

IL-1β, IL-6, and TNF-α concentrations in MH-S cell supernatants and mouse serum were measured using ELISA kits from MultiSciences Biotech (Hangzhou, China) according to the manufacturer’s instructions. Absorbance was recorded at 450 nm, and concentrations were calculated from the corresponding standard curves.

### 4.14. Western Blotting

Mouse lung tissue was homogenized in RIPA buffer containing a protease inhibitor cocktail, and protein concentrations were determined by BCA assay. Equal amounts of protein were separated by 8% or 12% SDS-PAGE and transferred to PVDF membranes. Membranes were blocked with 5% non-fat milk in TBST for 1.5 h at room temperature and incubated with primary antibodies overnight at 4 °C. After washing, membranes were incubated with HRP-conjugated secondary antibodies for 1 h at room temperature. Bands were detected by enhanced chemiluminescence and quantified in ImageJ (version 1.53t; National Institutes of Health, Bethesda, MD, USA). Primary antibodies were anti-NLRP3 (Proteintech, Wuhan, China, Cat. No. 19771-1-AP; 1:1000), anti-ASC (Proteintech, Cat. No. 10500-1-AP; 1:5000), anti-caspase-1 (Proteintech, Cat. No. 22915-1-AP; 1:1000), anti-GSDMD/GSDMD-N (Wanleibio, Shenyang, China, Cat. No. WL04008; 1:1000), and anti-β-actin (Proteintech, Cat. No. 60008-1-Ig; 1:5000). In accordance with manufacturer validation for murine tissue, the ~45 kDa band detected by the caspase-1 antibody was quantified as pro-caspase-1, and the ~30 kDa cleavage band detected by the GSDMD antibody was quantified as GSDMD-N. To compare all six experimental groups side by side on the same blot without inter-membrane transfer variability, three mice per group were randomly selected in a blinded manner from the ten mice in each group, and one lung sample from each of the six groups was loaded per 6-lane gel across three independent membranes (*n* = 3 independent biological replicates per group). Target band intensities were normalized to β-actin, whose band intensity remained stable across groups.

### 4.15. Quantitative PCR

Total RNA was isolated from MH-S cells and mouse lung tissue using the RNA/Protein Extraction Kit (Beyotime Biotechnology, Shanghai, China). cDNA was synthesized with the All-in-one First Strand cDNA Synthesis Kit III (Seven Biotech, Beijing, China), and qPCR was performed using 2× SYBR Green qPCR MasterMix II (Seven Biotech). Relative expression was calculated using the 2^−ΔΔCt^ method [40], with *Actb* (β-actin) as the endogenous reference gene; *Actb* cycle threshold (Ct) values showed no significant variation across experimental groups in either MH-S cells or lung tissue. Primer sequences are listed in Table 4.

### 4.16. Statistical Analysis

Data are presented as mean ± standard deviation (SD). Cellular assays comprised three independent biological experiments (*n* = 3) performed on separate days, with technical replicate wells averaged within each independent run prior to statistical analysis so that statistical tests were conducted strictly on independent biological units. Animal analyses included 10 independent mice per group (*n* = 10), except for Western blot densitometry, which included three randomly selected independent mice per group (*n* = 3). Prior to parametric testing, data normality and homogeneity of variance were assessed using the Shapiro–Wilk test and the Brown–Forsythe test, respectively, in GraphPad Prism 8.0 (GraphPad Software, San Diego, CA, USA). Group differences were analyzed by one-way analysis of variance (ANOVA) followed by Tukey’s multiple comparisons post hoc test. *p* < 0.05 was considered statistically significant.

### 4.17. Conceptual Illustration

Figure 12 provides a conceptual overview of the chemical profiling and cellular and animal findings. Data visualization and figure plotting were performed in Python (version 3.10; Python Software Foundation, Wilmington, DE, USA), and the final schematic and figures were prepared and reviewed by the authors.

## 5. Conclusions

Prophylactic administration of MEA suppressed LPS-induced inflammatory mediator production and DCFH-DA-detectable intracellular oxidative fluorescence in MH-S alveolar macrophages and attenuated histological lung injury, pulmonary edema, and circulating cytokines in an intraperitoneal LPS-induced mouse model of ALI. These protective effects were accompanied by decreased expression of *Nlrp3*, *Pycard*, *Casp1*, and *Gsdmd* mRNA as well as lower lung protein levels of NLRP3, ASC, pro-caspase-1, and ~30 kDa GSDMD-N. Together, these findings support the preventive anti-inflammatory activity of this Dong ethnomedicine preparation and provide a foundation for further constituent-level and functional mechanistic investigations.

## Figures and Tables

**Table 1:** Constituents of MEA confirmed with reference standards by UHPLC-Q-Exactive Orbitrap MS.

No.	tR (min)	Adduct	Theoretical *m*/*z*	Measured *m*/*z*	Error (ppm)	Formula	MS/MS Fragments (*m*/*z*, Relative Abundance %)	Identification
1	1.68	[M − H]^−^	169.01424	169.01312	−6.67	C_7_H_6_O_5_	125.0230 (100)	Gallic acid
2	6.82	[M + H]^+^/[M − H]^−^	579.14970/577.13515	579.14911/577.13464	−1.02/ −0.88	C_30_H_26_O_12_	289.0716 (100), 125.0232 (83), 407.0766 (69)	Procyanidin B1
3	7.45	[M + H]^+^/[M − H]^−^	291.08631/289.07176	291.08582/289.07144	−1.70/ −1.11	C_15_H_14_O_6_	109.0281 (100), 123.0438 (78), 125.0230 (50)	Catechin
4	12.76	[M − H]^−^	303.05102	303.05075	−0.91	C_15_H_12_O_7_	125.0231 (100), 175.0390 (17), 151.0026 (12)	Taxifolin
5	13.01	[M + H]^+^/[M − H]^−^	443.09727/441.08271	443.09689/441.08231	−0.87/ −0.93	C_22_H_18_O_10_	169.0131 (100), 289.0715 (36), 125.0231 (29)	Epicatechin gallate
6	13.09	[M − H]^−^	300.99899	300.99869	−1.00	C_14_H_6_O_8_	229.0135 (100), 201.0185 (63), 185.0238 (60)	Ellagic acid
7	14.04	[M − H]^−^	435.12967	435.12912	−1.26	C_21_H_24_O_10_	167.0339 (100), 273.0766 (76), 125.0231 (11)	Phlorizin
8	16.30	[M − H]^−^	301.03537	301.03519	−0.62	C_15_H_10_O_7_	151.0026 (100), 107.0125 (45), 178.9975 (15)	Quercetin
9	17.71	[M + H]^+^/[M − H]^−^	273.07575/271.06119	273.07544/271.06104	−1.14/ −0.58	C_15_H_12_O_5_	119.0489 (100), 107.0125 (45), 151.0026 (39)	Naringenin
10	17.82	[M − H]^−^	273.07684	273.07666	−0.68	C_15_H_14_O_5_	123.0439 (100), 119.0489 (75), 167.0339 (55)	Phloretin
11	19.09	[M − H]^−^	649.39572	649.39435	−2.11	C_36_H_58_O_10_	487.3425 (100)	Rosamultin
12	24.66	[M + H]^+^/[M − H]^−^	473.36253/471.34798	473.36209/471.34726	−0.94/ −1.53	C_30_H_48_O_4_	—	Pomolic acid
13	26.40	[M + H]^+^/[M − H]^−^	457.36762/455.35306	457.36670/455.35266	−2.02/ −0.90	C_30_H_48_O_3_	—	Ursolic acid

Notes: All 13 compounds were confirmed by comparison of retention time and MS/MS spectra with reference standards analyzed under identical conditions. —, no informative fragment ions. Abbreviations: MEA, ethyl acetate extract of Madeng’ai; MS/MS, tandem mass spectrometry; tR, retention time; UHPLC, ultra-high-performance liquid chromatography.

**Table 2:** Candidate MEA compounds meeting the TCMSP screening thresholds.

Compound	Class	Formula	MW	No. in Table S1	Identification	Role in This Study
β-Sitosterol	Phytosterol	C_29_H_50_O	414.71	77	Tentative (MS/MS)	Candidate compound in network analysis
Betulinic acid	Triterpenoid	C_30_H_48_O_3_	456.71	76	Tentative (MS/MS)	Candidate compound in network analysis
Kaempferol	Flavonoid	C_15_H_10_O_6_	286.24	74	Tentative (MS/MS)	Representative ligand for NFKB1 docking
Isorhamnetin	Flavonoid	C_16_H_12_O_7_	316.26	75	Tentative (MS/MS)	Representative ligand for EGFR docking
Ellagic acid	Tannin-related polyphenol	C_14_H_6_O_8_	302.19	51	Reference standard	Representative ligand for caspase-1 docking
Quercetin	Flavonoid	C_15_H_10_O_7_	302.24	61	Reference standard	Representative ligand for NLRP3 docking

Notes: Candidate compounds met oral bioavailability ≥ 30% and drug-likeness ≥ 0.18 in TCMSP. MW, molecular weight (g/mol) calculated from the molecular formula. Abbreviations: EGFR, epidermal growth factor receptor; MEA, ethyl acetate extract of Madeng’ai; MS/MS, tandem mass spectrometry; NFKB1, nuclear factor kappa B subunit 1; NLRP3, NOD-like receptor family pyrin domain-containing 3; TCMSP, Traditional Chinese Medicine Systems Pharmacology Database and Analysis Platform.

**Table 3:** Hub targets and their principal biological functions.

Gene Symbol	Full Name	Main Biological Role
*AKT1*	AKT serine/threonine kinase 1	PI3K-Akt survival and inflammatory signaling
*EGFR*	Epidermal growth factor receptor	Epithelial injury response; MAPK/PI3K-Akt activation
*ESR1*	Estrogen receptor 1	Immune modulation and transcriptional regulation
*IL1B*	Interleukin 1 beta	Inflammasome-related pro-inflammatory cytokine
*IL6*	Interleukin 6	Acute inflammatory cytokine; JAK-STAT signaling
*NFKB1*	Nuclear factor kappa B subunit 1	Inflammatory transcription and inflammasome priming
*SRC*	SRC proto-oncogene, non-receptor tyrosine kinase	Receptor-linked kinase and inflammatory signaling
*STAT3*	Signal transducer and activator of transcription 3	Cytokine signaling and inflammatory repair
*TNF*	Tumor necrosis factor	Central cytokine in inflammatory amplification

Abbreviations: AKT1, AKT serine/threonine kinase 1; EGFR, epidermal growth factor receptor; ESR1, estrogen receptor 1; IL1B, interleukin 1 beta; IL6, interleukin 6; NFKB1, nuclear factor kappa B subunit 1; SRC, SRC proto-oncogene; STAT3, signal transducer and activator of transcription 3; TNF, tumor necrosis factor.

**Table 4:** Primer sequences used for RT-qPCR.

Gene	Forward Primer (5′–3′)	Reverse Primer (5′–3′)
*Nlrp3*	TCACAACTCGCCCAAGGAGAA	AAGAGACCACGGCAGAAGCTAG
*Pycard* (ASC)	CTGCTCAGATACAGCCAGAAC	CTGTCCTTCAGTCAGCACACTG
*Gsdmd*	GGTGCTTGACTCTGGGAACTG	GCTGCTTTGACAGCACCGTTGT
*Casp1*	GGCACATTTCCAGGACTGACTG	GCAAGACGTGTACGAGTGGTTG
*Actb* (β-actin)	CATGCTGACAGATGCAGAAGG	TGCTGGAAGGTGGACAGTGAGG

Abbreviations: ASC, apoptosis-associated speck-like protein containing a CARD; RT-qPCR, reverse transcription quantitative polymerase chain reaction.

## Data Availability

The data supporting the findings of this study are available from the corresponding authors upon reasonable request. Uncropped Western blot images are provided separately as source data for editorial review.

## Supplementary Materials

The following supporting information can be downloaded at https://www.mdpi.com/article/10.3390/ijms27219430/s1.

## Abbreviations

ALI, acute lung injury; ANOVA, analysis of variance; ARDS, acute respiratory distress syndrome; ASC, apoptosis-associated speck-like protein containing a CARD; CCK-8, Cell Counting Kit-8; DCFH-DA, 2′,7′-dichlorodihydrofluorescein diacetate; DEX, dexamethasone; DL, drug-likeness; ELISA, enzyme-linked immunosorbent assay; GO, Gene Ontology; GSDMD, gasdermin D; GSDMD-N, N-terminal fragment of gasdermin D; H&E, hematoxylin and eosin; KEGG, Kyoto Encyclopedia of Genes and Genomes; LPS, lipopolysaccharide; MDA, Madeng’ai; MEA, ethyl acetate fraction of the ethanolic extract of Madeng’ai; NLRP3, NOD-like receptor family pyrin domain-containing 3; OB, oral bioavailability; PPI, protein–protein interaction; ROS, reactive oxygen species; RT-qPCR, reverse transcription quantitative polymerase chain reaction; UHPLC-MS, ultra-high-performance liquid chromatography–mass spectrometry.
