# Supplementary material for: Madeng’ai Ethyl Acetate Extract Attenuates Lipopolysaccharide-Induced Inflammation and Acute Lung Injury
Source: Int J Mol Sci. 2026 Oct 22;27(21):9430. doi: 10.3390/ijms27219430 (PMC13763553; doi:10.3390/ijms27219430)
Supplement: Supplementary file 1 [file ijms-27-09430-s001.zip › ijms-4632117-supplementary.pdf]

Supplementary Table S1. Constituents of MEA identified by UHPLC-Q-Exactive Orbitrap MS

No.	tR (min)	Adduct	Theoretical m/z	Measured m/z	Error (ppm)	Formula	MS/MS fragments (m/z, relative abundance %)	Identification	Class
1	1.34	$[M-H]^-$	331.06706	331.06696	-0.33	$C_{13}H_{16}O_{10}$	MS2[331]: 169.0132(100), 125.0231(56), 211.0242(44)	1-O-Galloylglucose	Tannin
2	1.68†	$[M-H]^-$	169.01424	169.01312	-6.67	$C_7H_6O_5$	MS2[169]: 125.0230(100)	Gallic acid	Organic acid
3	2.53	$[M-H]^-$	331.06706	331.06683	-0.72	$C_{13}H_{16}O_{10}$	MS2[331]: 169.0132(100), 125.0231(78)	3-O-Galloylglucose	Tannin
4	2.81	$[M-H]^-$	483.07802	483.07764	-0.80	$C_{20}H_{20}O_{14}$	MS2[483]: 169.0131(100), 331.0668(16), 313.0564(12), 125.0232(10)	Digalloylglucose	Tannin
5	3.18	$[M-H]^-$	153.01933	153.01933	-7.46	$C_7H_6O_4$	MS2[153]: 108.0204(100), 109.0282(89)	Protocatechuic acid	Organic acid
6	3.80	$[M-H]^-$	345.08271	345.08231	-1.19	$C_{14}H_{18}O_{10}$	MS2[345]: 169.0132(100), 151.0025(78), 125.0232(54)	Methyl 6-O-galloylglucopyranoside	Tannin
7	3.89	$[M-H]^-$	483.07802	483.07764	-0.80	$C_{20}H_{20}O_{14}$	MS2[483]: 169.0132(100), 211.0241(94), 271.0457(86), 313.0562(30), 125.0231(12)	Digalloylglucose	Tannin
8	5.58	$[M-H]^-$	633.07333	633.07318	-0.25	$C_{27}H_{22}O_{18}$	MS2[633]: 300.9987(100), 275.0205(14)	GalloyHHDP-hexoside	Tannin
9	5.70	$[M-H]^-$	451.12458	451.12430	-0.63	$C_{21}H_{24}O_{11}$	MS2[451]: 289.0716(100), 245.0814(37), 125.0231(11), 205.0496(10), 203.0705(10)	Catechin-5-O-glucoside	Flavonoid
10	5.87	$[M-H]^-$	483.07802	483.07776	-0.56	$C_{20}H_{20}O_{14}$	MS2[483]: 169.0132(100), 271.0458(49), 211.0241(43), 313.0572(12), 125.0232(10)	Digalloylglucose	Tannin
11	6.21	$[M-H]^-$	183.02989	183.02902	-4.79	$C_8H_8O_5$	MS2[183]: 140.0102(100), 111.0075(57), 124.0152(41), 136.9868(39)	Methyl gallate	Organic acid
12	6.38	$[M-H]^-$	451.12458	451.12427	-0.70	$C_{21}H_{24}O_{11}$	MS2[451]: 137.0232(100), 125.0231(47), 151.0389(30), 289.0720(3)	Catechin-7-O-glucoside	Flavonoid
13	6.79	$[M-H]^-$	483.07802	483.07791	-0.25	$C_{20}H_{20}O_{14}$	MS2[483]: 169.0132(100), 271.0457(43), 211.0239(32), 125.0231(8)	Digalloylglucose	Tannin
14	6.82†	$[M-H]^-$	577.13515	577.13464	-0.88	$C_{30}H_{26}O_{12}$	MS2[577]: 289.0716(100), 125.0232(83), 407.0766(69), 245.0813(13)	Procyanidin B1	Flavonoid
15	7.15	$[M-H]^-$	483.07802	483.07767	-0.74	$C_{20}H_{20}O_{14}$	MS2[483]: 169.0132(100), 271.0457(47), 211.0241(38), 313.0566(13), 125.0232(9)	Digalloylglucose	Tannin
16	7.21	$[M-H]^-$	577.13515	577.13464	-0.88	$C_{30}H_{26}O_{12}$	MS2[577]: 289.0716(100), 407.0967(88), 125.0231(72), 245.0814(14)	Procyanidin B-type	Flavonoid
17	7.45†	$[M-H]^-$	289.07176	289.07144	-1.11	$C_{15}H_{14}O_6$	MS2[289]: 109.0281(100), 123.0438(78), 125.0230(50), 151.0388(38), 137.0231(32), 203.0702(26)	Catechin	Flavonoid
18	7.58	$[M-H]^-$	465.10384	465.10327	-1.25	$C_{21}H_{22}O_{12}$	MS2[465]: 285.0401(100), 125.0231(49), 275.0558(23), 177.0182(21), 303.0507(14)	Taxifolin-7-O-glucoside	Flavonoid
19	7.80	$[M-H]^-$	483.07802	483.07745	-1.20	$C_{20}H_{20}O_{14}$	MS2[483]: 211.0240(100), 271.0456(97), 169.0131(79), 313.0565(31), 331.0674(5)	Digalloylglucose	Tannin

No.	tR (min)	Adduct	Theoretical m/z	Measured m/z	Error (ppm)	Formula	MS/MS fragments (m/z, relative abundance %)	Identification	Class
20	8.02	$[M-H]^-$	305.06667	305.06635	-1.07	$C_{15}H_{14}O_7$	MS2[305]: 125.0231(100), 161.0233(30), 109.0282(28), 137.0323(25)	Gallocatechin	Flavonoid
21	8.19	$[M-H]^-$	865.19853	865.19806	-0.55	$C_{45}H_{38}O_{18}$	MS2[865]: 287.0557(100), 577.1339(60), 125.0230(52), 425.0871(47), 413.0883(44), 289.0716(42)	Procyanidin C-type	Flavonoid
22	8.35	$[M-H]^-$	483.07802	483.07758	-0.93	$C_{20}H_{20}O_{14}$	MS2[483]: 271.0457(100), 169.0132(78), 313.0565(42), 311.0242(42), 125.0232(10)	Digalloylglucose	Tannin
23	8.66	$[M-H]^-$	633.07333	633.07281	-0.83	$C_{27}H_{22}O_{18}$	MS2[633]: 300.9987(100), 275.0196(10)	GalloyHHDP-hexoside	Tannin
24	8.83	$[M-H]^-$	865.19853	865.19806	-0.55	$C_{45}H_{38}O_{18}$	MS2[865]: 287.0560(100), 125.0231(58), 425.0871(54), 289.0716(49)	Procyanidin C-type	Flavonoid
25	9.19	$[M-H]^-$	483.07802	483.07751	-1.07	$C_{20}H_{20}O_{14}$	MS2[483]: 169.0132(100), 125.0231(78), 331.0669(70), 313.0565(23)	Digalloylglucose	Tannin
26	9.23	$[M-H]^-$	1153.26192	1153.26099	-0.81	$C_{60}H_{50}O_{24}$	MS2[1153]: 575.1190(100), 577.1343(63), 865.1983(48), 863.1829(40), 287.0558(34)	Cinnamtannin A2	Flavonoid
27	9.28	$[M-H]^-$	635.08898	635.08844	-0.86	$C_{27}H_{24}O_{18}$	MS2[635]: 165.0132(100), 465.0669(42), 313.0562(29), 483.0779(14), 125.0229(6)	Trigalloylglucose	Tannin
28	9.42	$[M-H]^-$	633.07333	633.07306	-0.44	$C_{27}H_{22}O_{18}$	MS2[633]: 300.9987(100), 463.0525(15), 275.0190(8)	GalloyHHDP-hexoside	Tannin
29	9.67	$[M-H]^-$	865.19853	865.19836	-0.21	$C_{45}H_{38}O_{18}$	MS2[865]: 287.0558(100), 425.0874(47), 125.0231(46), 577.1349(46)	Procyanidin C-type	Flavonoid
30	9.70	$[M-H]^-$	635.08898	635.08905	0.10	$C_{27}H_{24}O_{18}$	MS2[635]: 465.0669(100), 169.0132(90), 313.568(50), 483.0776(44), 211.0238(9), 125.0231(8)	Trigalloylglucose	Tannin
31	9.96	$[M-H]^-$	483.07802	483.07767	-0.74	$C_{20}H_{20}O_{14}$	MS2[483]: 169.0131(100), 313.0563(30), 125.0232(14), 331.0662(7)	Digalloylglucose	Tannin
32	10.46	$[M-H]^-$	635.08898	635.08844	-0.86	$C_{27}H_{24}O_{18}$	MS2[635]: 169.0132(100), 465.0673(54), 483.0778(35), 313.0565(28), 125.0232(10)	Trigalloylglucose	Tannin
33	10.46	$[M-H]^-$	785.08429	785.08380	-0.63	$C_{34}H_{26}O_{22}$	MS2[785]: 300.9989(100), 249.0397(20), 275.0201(17), 483.0780(16), 313.0562(8), 169.0132(8)	digalloyl-HHDP-glucose	Tannin
34	10.60	$[M-H]^-$	289.07176	289.07150	-0.90	$C_{15}H_{14}O_6$	MS2[289]: 109.0282(100), 123.0439(77), 125.0231(50), 151.0388(36), 137.0231(28), 203.0706(23)	Epicatechin	Flavonoid
35	10.63	$[M-H]^-$	577.13515	577.13464	-0.88	$C_{30}H_{26}O_{12}$	MS2[577]: 125.0231(100), 289.0715(90), 407.0766(64), 245.0814(13)	Procyanidin B-type	Flavonoid
36	10.74	$[M-H]^-$	865.19853	865.19775	-0.91	$C_{45}H_{38}O_{18}$	MS2[865]: 287.0559(100), 125.0230(70), 289.0714(44), 425.0867(37), 577.1362(31)	Procyanidin C-type	Flavonoid
37	11.10	$[M-H]^-$	197.04554	197.04468	-4.40	$C_9H_{10}O_5$	MS2[197]: 125.0231(100), 140.0102(31), 111.0073(22), 136.9866(14), 169.0126(8)	Ethyl gallate	Organic acid

No.	tR (min)	Adduct	Theoretical m/z	Measured m/z	Error (ppm)	Formula	MS/MS fragments (m/z, relative abundance %)	Identification	Class
38	11.24	$[M-H]^-$	635.08898	635.08868	-0.48	$C_{27}H_{24}O_{18}$	MS2[635]: 465.0669(100), 169.0131(44), 313.0563(34), 483.0787(18), 211.0242(7)	Trigalloylglucose	Tannin
39	11.35	$[M-H]^-$	729.14610	729.14569	-0.57	$C_{37}H_{30}O_{16}$	MS2[729]: 407.0765(100), 287.0558(69), 289.0715(64), 125.0231(63), 169.0131(60), 425.0872(60), 577.1351(41)	Procyanidin B-Type -O-gallate	Flavonoid
40	11.44	$[M-H]^-$	865.19853	865.19800	-0.62	$C_{45}H_{38}O_{18}$	MS2[865]: 287.0555(100), 125.0230(85), 289.0723(49), 577.1342(39)	Procyanidin C-type	Flavonoid
41	11.71	$[M-H]^-$	729.14610	729.14569	-0.57	$C_{37}H_{30}O_{16}$	MS2[729]: 407.0766(100), 289.0715(67), 125.0231(46), 425.0876(41), 169.0134(38)	Procyanidin B-Type -O-gallate	Flavonoid
42	11.76	$[M-H]^-$	577.13515	577.13452	-1.09	$C_{30}H_{26}O_{12}$	MS2[577]: 289.0716(100), 125.0231(94), 407.0766(64), 245.0813(14)	Procyanidin B-type	Flavonoid
43	12.12	$[M-H]^-$	441.08271	441.08231	-0.93	$C_{22}H_{18}O_{10}$	MS2[441]: 289.0715(100), 245.0814(52), 125.0231(18), 205.0498(15), 203.0706(15)	Catechin gallate	Flavonoid
44	12.34	$[M-H]^-$	575.11949	575.11951	0.02	$C_{30}H_{24}O_{12}$	MS2[575]: 125.0231(100), 285.0403(69), 289.0714(67), 287.0559(22), 407.0771(19)	Procyanidin A-type	Flavonoid
45	12.62	$[M-H]^-$	787.09994	787.10010	0.20	$C_{34}H_{28}O_{22}$	MS2[787]: 617.0782(100), 465.0665(45), 169.0133(33), 635.0885(16), 313.0567(5)	1,2,3,6-Tetragalloylglucose	Tannin
46	12.76†	$[M-H]^-$	303.05102	303.05075	-0.91	$C_{15}H_{12}O_7$	MS2[303]: 125.0231(100), 175.0390(17), 151.0026(12)	Taxifolin	Flavonoid
47	12.87	$[M-H]^-$	575.11949	575.11969	0.33	$C_{30}H_{24}O_{12}$	MS2[575]: 285.0403(100), 125.0232(96), 289.0717(73), 407.0769(31), 287.0557(27)	Procyanidin A-type	Flavonoid
48	12.87	$[M-H]^-$	477.14023	477.13989	-0.72	$C_{23}H_{26}O_{11}$	MS2[477]: 169.0131(100), 313.0563(55), 151.0026(19), 125.0231(12)	Lindleyin	Tannin
49	13.01†	$[M-H]^-$	441.08271	441.08231	-0.93	$C_{22}H_{18}O_{10}$	MS2[441]: 169.0131(100), 289.0715(36), 125.0231(29), 245.0813(19), 137.0232(6), 205.0498(6)	Epicatechin gallate	Flavonoid
50	13.03	$[M-H]^-$	729.14610	729.14581	-0.41	$C_{37}H_{30}O_{16}$	MS2[729]: 407.0770(100), 169.0131(73), 125.0232(68), 289.0716(61), 287.0559(57)	Procyanidin B-Type -O-gallate	Flavonoid
51	13.09†	$[M-H]^-$	300.99899	300.99869	-1.00	$C_{14}H_6O_8$	MS2[301]: 229.0135(100), 201.0185(63), 185.0238(60), 283.9955(58), 257.0085(47), 245.0085(41)	Ellagic acid	Tannin
52	13.23	$[M-2H]^{2-}$	469.05181	469.05151	1.87	$C_{41}H_{32}O_{26}$	MS2[469]: 169.0132(100), 125.0231(31)	1,2,3,4,6-Pentagalloylglucose	Tannin
53	13.77	$[M-H]^-$	447.05689	447.05655	-0.78	$C_{20}H_{16}O_{12}$	MS2[447]: 315.0143(100), 299.9908(37)	Methylellagic acid-O-rhamnoside	Tannin
54	13.79	$[M-H]^-$	463.08819	463.08789	-0.67	$C_{21}H_{20}O_{12}$	MS2[463]: 151.0024(100), 287.0557(49), 125.0231(20)	Eriodictyol-O-glucuronide	Flavonoid
55	13.93	$[M-H]^-$	575.11949	575.11926	-0.42	$C_{30}H_{24}O_{12}$	MS2[575]: 394.0689(100), 125.0231(37), 137.0232(31), 287.0558(30), 407.0768(25)	Procyanidin A-type	Flavonoid
56	14.04†	$[M-H]^-$	435.12967	435.12912	-1.26	$C_{21}H_{24}O_{10}$	MS2[435]: 167.0339(100), 273.0766(76), 125.0231(11), 123.0439(5)	Phlorizin	Flavonoid
57	14.10	$[M-H]^-$	447.05689	447.05646	-0.98	$C_{20}H_{16}O_{12}$	MS2[447]: 315.0145(100), 399.9909(26)	Methylellagic acid-O-rhamnoside	Tannin

No.	tR (min)	Adduct	Theoretical m/z	Measured m/z	Error (ppm)	Formula	MS/MS fragments (m/z, relative abundance %)	Identification	Class
58	14.18	[M−H]⁻	477.06746	477.06699	-0.99	$C_{21}H_{18}O_{13}$	MS2[477]: 301.0351(100), 178.9974(10), 151.0025(10)	quercetin 3-O-glucuronide	Flavonoid
59	14.54	[M−H]⁻	315.01464	315.01459	-0.16	$C_{15}H_{8}O_{8}$	MS2[315]: 299.9908(100)	methylellagic acid	Tannin
60	15.48	[M−H]⁻	287.05611	287.05588	-0.81	$C_{15}H_{12}O_{6}$	MS2[287]: 135.0439(100), 151.0025(24), 107.0125(22)	Eriodictyol	Flavonoid
61	16.30†	[M−H]⁻	301.03537	301.03519	-0.62	$C_{15}H_{10}O_{7}$	MS2[301]: 151.0026(100), 107.0125(45), 178.9975(15)	Quercetin	Flavonoid
62	16.93	[M−H]⁻	329.03029	329.03015	-0.43	$C_{16}H_{10}O_{8}$	MS2[329]: 298.9831(100), 270.9882(37), 314.0067(25), 299.9853(5)	dimethylellagic acid	Tannin
63	17.71†	[M−H]⁻	271.06119	271.06104	-0.58	$C_{15}H_{12}O_{5}$	MS2[271]: 119.0489(100), 107.0125(45), 151.0026(39)	Naringenin	Flavonoid
64	17.82†	[M−H]⁻	273.07684	273.07666	-0.68	$C_{15}H_{14}O_{5}$	MS2[273]: 123.0439(100), 119.0489(75), 167.0339(55), 125.0232(28), 151.0024(16), 107.0126(9)	Phloretin	Flavonoid
65	19.09†	[M−H]⁻	649.39572	649.39435	-2.11	$C_{36}H_{58}O_{10}$	MS2[649]: 487.3425(100)	Rosamultin	Triterpenoid
66	19.09	[M−H]⁻	695.40120	695.40063	-0.82	$C_{37}H_{60}O_{12}$	MS2[695]: 487.3426(100)	Momordicoside E isomer	Triterpenoid
67	20.38	[M−H]⁻	693.38555	693.38513	-0.61	$C_{37}H_{58}O_{12}$	MS2[693]: 485.3268(100)	Acanthosessilioside C isomer	Triterpenoid
68	21.99	[M−H]⁻	485.32724	485.32663	-1.27	$C_{30}H_{46}O_{5}$	MS2[485]: 467.3156(100), 407.2940(59)	rosamultic acid isomer	Triterpenoid
69	22.82	[M−H]⁻	487.34289	487.34235	-1.12	$C_{30}H_{48}O_{5}$	MS2[487]: 469.3320(100), 423.3287(30), 425.3418(28)	Tormentic acid	Triterpenoid
70	23.81	[M−H]⁻	485.32724	485.32672	-1.09	$C_{30}H_{46}O_{5}$	MS2[485]: 467.3162(100), 423.3268(10)	rosamultic acid isomer	Triterpenoid
71	24.52	[M−H]⁻	469.33233	469.33148	-1.82	$C_{30}H_{46}O_{4}$	MS2[469]: 407.2941(100), 451.3238(56), 423.3255(47), 405.2795(15)	coleonolic acid	Triterpenoid
72	24.66†	[M−H]⁻	471.34798	471.34726	-1.53	$C_{30}H_{48}O_{4}$	MS2[471]: -	Pomolic acid	Triterpenoid
73	26.40†	[M−H]⁻	455.35306	455.35266	-0.90	$C_{30}H_{48}O_{3}$	MS2[455]: -	Ursolic acid	Triterpenoid
74	16.50	[M−H]⁻	285.04046	285.04012	-1.19	$C_{15}H_{10}O_{6}$	MS2[285]: 153.0182(100), 177.0185(45), 257.0455(20), 229.0501(15)	Kaempferol	Flavonoid
75	17.12	[M−H]⁻	315.05102	315.05065	-1.17	$C_{16}H_{12}O_{7}$	MS2[315]: 300.0275(100), 299.0195(60), 271.0245(30), 151.0025(20)	Isorhamnetin	Flavonoid
76	25.80	[M−H]⁻	455.35306	455.35266	-0.88	$C_{30}H_{48}O_{3}$	MS2[455]: 437.3421(100), 411.3619(45), 311.2715(30), 255.2325(20)	Betulinic acid	Triterpenoid
77	28.45	[M+H−H2O]⁺	413.37888	413.37845	-1.04	$C_{29}H_{50}O$	MS2[413]: 398.3541 (100), 161.1325(45), 147.1168(30)	β-Sitosterol	Phytosterol

Notes: †Confirmed by comparison of retention time and MS/MS spectra with reference standards; all other compounds were tentatively assigned from accurate mass and MS/MS fragmentation.

Abbreviations: MEA, ethyl acetate extract of Madeng'ai; MS/MS, tandem mass spectrometry; tR, retention time; UHPLC, ultra-high-performance liquid chromatography.
